# Loss of amyloid precursor protein exacerbates early inflammation in Niemann-Pick disease type C

**DOI:** 10.1186/s12974-019-1663-5

**Published:** 2019-12-17

**Authors:** Samuel D. Shin, Alexandra Shin, Karina Mayagoitia, Lorraine Siebold, Marsilio Rubini, Christopher G. Wilson, Denise L. Bellinger, Salvador Soriano

**Affiliations:** 1 Department of Pathology and Human Anatomy, School of Medicine, Loma Linda University, 24785 Stewart St, Loma Linda, California USA; 2 Department of Biological Sciences, California Baptist University, Riverside, California USA; 3 Lawrence D. Longo Center for Perinatal Biology, Loma Linda University School of Medicine, Loma Linda, California USA

**Keywords:** Niemann-Pick disease type C, Neurodegeneration, Neuroinflammation, Gene Expression, Amyloid precursor protein

## Abstract

**Background:**

Niemann-Pick disease type C (NPC) is a progressive neurodegenerative condition that results in early fatality. NPC is inherited in an autosomal recessive pattern from mutations in *NPC1* or *NPC2* genes. The etiology of NPC is poorly defined. In that regard, neuroinflammation occurs early in the disease and we have recently unveiled an atypical pattern of interferon signaling in pre-symptomatic *Npc1*^−/−^ mice, with microglial activation, anti-viral response, activation of antigen-presenting cells, and activation and chemotaxis of T lymphocytes as the key affected pathologic pathways. Furthermore, IP-10/CXCL10, a potent IFN-γ-responsive cytokine, was identified as the potential mediator of these early inflammatory abnormalities. Here, we asked whether this aberrant signaling may be exacerbated by the loss of amyloid precursor protein (APP) function, a loss known to shorten lifespan and accelerate neurodegeneration in *Npc1*^−/−^ mice.

**Methods:**

We carried out genome-wide comparative transcriptome analyses of pre-symptomatic *Npc1*^*+/+*^*/App*^*+/+*^, *Npc1*^−/−^*/App*^*+/+*^, *Npc1*^*+/+*^*/App*^−/−^, and *Npc1*^−/−^*/App*^−/−^ mouse cerebella to identify biological pathways in the NPC brain further affected by the loss of APP. *Gene Set Enrichment Analysis* and *Ingenuity Pathway Analysis* were utilized for molecular mapping and functional upstream pathway analyses of highly differentially expressed genes. We simultaneously measured the expression of 32 inflammatory cytokines and chemokines in the cerebella from these mice, including those identified in our genome-wide analyses. Finally, we used immunohistochemistry to measure T cell infiltration in the cerebellum.

**Results:**

Expression of IFN-γ- and IFN-α-responsive genes in pre-symptomatic *Npc1*^−/−^*/App*^−/−^ cerebella is upregulated compared with *Npc1*^−/−^*/App*^*+/+*^ mice, compounding the dysregulation of microglial activation, anti-viral response, activation of antigen-presenting cells, and T-lymphocyte activation and chemotaxis pathways present in the NPC brain. Multiplex protein analysis further showed elevated expression of IP-10/CXCL10, a potent downstream effector of IFN-γ, as well as RANTES/CCL5, eotaxin/CCL11 and IL-10, prior to symptomatic onset in *Npc1*^−/−^*/App*^−/−^ cerebella, compared with *Npc1*^−/−^*/App*^*+/+*^mice. In the terminal disease stage, loss of APP caused pleiotropic differential expression of the vast majority of cytokines evaluated. Finally, we present evidence of T cell infiltration in *Npc1*^−/−^*/App*^−/−^ cerebella.

**Conclusions:**

Loss of APP exacerbates the pathogenic neuroinflammation that occurs prior to symptomatic onset in the NPC brain. These findings shed new light on the function of APP as a cytoprotective modulator in the CNS, offering potential evidence-based therapies against NPC.

## Background

Niemann-Pick disease type C (NPC) is a neurodegenerative disease inherited in an autosomal recessive pattern [1], with mutations in the NPC1 gene accounting for approximately 95% of all reported cases and the remaining 5% of the cases resulting from mutations in the NPC2 gene. While NPC affects all cell types in the body, the main disease feature is the progressive neurodegeneration that results in premature death [1]. Currently, there is no cure or effective therapy available for NPC and the mechanistic etiology of neurodegeneration remains poorly defined [1–4]. Functionally, the biological roles of NPC1and NPC2 are not well defined, a shortcoming that has hampered our understanding of the mechanistic etiology of the disease. At the cellular level, the disease phenotype is broad, affecting multiple functions, such as endosomal lipid accumulation, calcium dysregulation, neuroinflammation, mitochondrial dysfunction, amyloid peptide Aβ accumulation and tau hyperphosphorylation and aggregation. Nevertheless, the pathogenic hierarchy of these cellular dysfunctions remains unresolved. Several laboratories, including ours, have demonstrated that neuroinflammation occurs early in the disease. We have specifically shown an atypical activation pattern of interferon downstream signaling that involves both IFN-γ- and IFN-α-responsive genes in pre-symptomatic *Npc1*^−/−^ cerebella. Activation of IFN-γ and IFN-α responsive genes predicts abnormal microglial activation, anti-viral response, antigen-presenting cell and T-lymphocyte activation, and chemotaxis signaling prior to symptomatic onset [5]. Notably, IP-10/CXCL10 was the only significantly upregulated cytokine detected at this pre-symptomatic stage, suggesting that this effector of both IFN-γ- and IFN-α signaling could be a key early mediator of aberrant neuroinflammation in NPC.

Here, we asked whether the amyloid precursor protein (APP) plays a role in the early interferon-driven aberrant signaling observed in the pre-symptomatic NPC brain. APP is a disease modifier of Niemann-Pick disease type C (NPC). The loss of the *App* gene in the NPC mouse model BALB/cNctr-*Npc1*^*miN*^/J (*Npc1*^−/−^/*App*^−/−^) results in increased neuroinflammation marked by reactive astrocytosis, decreased neuromuscular function, accelerated neuronal death and shorter lifespan [6]. Thus, APP exerts a protective function in the NPC brain, consistent with the mounting evidence in support of its role as a neuronal stress modulator [7–14]. The specific nature of this neuroprotective role in NPC remains unknown; here, we reasoned that it may exert its protective function by modulating the inflammatory response of the CNS milieu. To explore that possibility, we carried out a comparative genome-wide transcriptome analysis of pre-symptomatic cerebellar tissue samples from *Npc1*^*+/+*^*/App*^*+/+*^, *Npc1*^*+/+*^*/App*^−/−^, *Npc1*^−/−^*/App*^*+/+*^, and *Npc1*^−/−^*/App*^−/−^ mice to identify NPC-altered genes and pathways further affected by the loss of APP. Furthermore, we carried out *in vivo* protein validation of the key pro-and anti-inflammatory cytokines and chemokines identified in our genome-wide analyses. Our results show that, in pre-symptomatic *Npc1*^−/−^*/App*^−/−^ cerebella, expression of IFN-γ- and IFN-α-responsive genes is significantly upregulated compared with *Npc1*^−/−^*/App*^*+/+*^ mice, compounding the dysregulation of microglial activation, anti-viral response, activation of antigen-presenting cells, and T-lymphocyte activation and chemotaxis pathways present in the latter. Multiplex analysis further showed elevated expression of IP-10/CXCL10, MIG/CXCL9, RANTES/CCL5, eotaxin/CCL11 and IL-10 prior to symptomatic onset in *Npc1*^−/−^*/App*^−/−^ cerebella compared with *Npc1*^−/−^*/App*^*+/+*^mice. In the terminal stage, loss of APP caused pleiotropic differential expression of the vast majority of cytokines evaluated. These findings add to the growing evidence in support of a cytoprotective role of APP in the brain and suggest that, in NPC, that role is mediated through the modulation of neuroinflammation.

## Methods

### Mice

This study was approved by Loma Linda University Institutional Animal Care and Use Committee (LLU #8170041 and LLU#8180006). *Npc1*^+/+^ and *Npc1*^−/−^ littermates were generated from a breeding colony of BALB/cNctr-*Npc1*^*miN*^/J from the Jackson Laboratory. In order to generate *Npc1*^+/+^ or *Npc1*^−/−^ mice that lack one or both *App* alleles, we crossed APP knockout mice (B6.129S7- App^*tm1Dbo*^/J mice *App*^−/−^ from the Jackson Laboratory) to generate breeders double heterozygous for NPC1 and APP (*Npc1*^+/−^/*App*^+/−^), backcrossed to homogenize their genetic backgrounds, as previously described by us [6]. The following genotypes were collected for multiplex protein analysis at pre-symptomatic and terminal stages: wild-type control (*Npc1*^+/+^/*App*^+/+^), APP knockout (*Npc1*^*+/+*^*/App*^−/−^), NPC1 knockout (*Npc1*^−/−^*/App*^*+/+*^), NPC1 knockout/APP heterozygous (*Npc1*^−/−^/*App*^+/−^), and NPC1/APP double knockout (*Npc1*^−/−^/*App*^−/−^) mice. Genotypes were determined as previously described [5, 6].

### Microarray hybridization and transcriptome analysis

Cerebellar samples of pre-symptomatic *Npc1*^*+/+*^*/App*^*+/+*^, *Npc1*^*+/+*^*/App*^−/−^, *Npc1*^−/−^*/App*^*+/+*^, and *Npc1*^−/−^*/App*^−/−^ *mice* were sent to GenUs (GenUs Biosystems, Northbrook, IL) for RNA processing and microarray hybridization. A separate v2 GE 4x44 microarray chip was used for each sample, for a total of 12 chips (n = 3 for each genotype; males between 3 to 6 weeks). For differentially expressed genes (DEGs) selection, both fold-change (absolute FC >1.5) and *p*-value (*p* < 0.05) cutoffs were used, as previously described [15]. To correct for multiple comparison, statistical significance between genotypes was determined by one-way analysis of variance (ANOVA) and a protected Tukey’s post hoc test with *p* < 0.05 considered significant. Next, the transcriptome was analyzed by the *Gene-Set Enrichment Analysis* software (GSEA, www.broadinstitute.org/gsea), as previously described [16] using c1.Hallmark database of the Molecular Signature Databases (MSigDB, http://software.broadinstitute.org/gsea/msigdb). Normalized enrichment score (NES) and false discovery rate (FDR) were calculated by the GSEA and enriched gene-sets displaying FDR < 0.25 were considered significant, as recommended by the software developers [16]. *Ingenuity Pathway Analysis* software (IPA, Qiagen, Redwood City CA) was utilized for molecular mapping and functional pathway analysis of the highly DEGs with priority in identifying the most affected pathways in early NPC.

### Multiplex cytokine/chemokine detection

The levels of 32 inflammatory cytokines and chemokines in the cerebella from *Npc1*^*+/+*^*/App*^*+/+*^, *Npc1*^*+/+*^*/App*^−/−^, *Npc1*^−/−^*/App*^*+/+*^, *Npc1*^−/−^*/App*^+/−^, and *Npc1*^−/−^*/App*^−/−^ mice were simultaneously analyzed using Milliplex 32-plex Mouse Cytokine/Chemokine Magnetic Bead Panel (Catalog# MCYTMAG-70 K-PX32, Millipore Sigma, Burlington MA) according to the manufacturer’s instructions. Cerebellar tissue was homogenized in protein extraction buffer (PBS, 0.05% Triton X, Halt^TM^ Protease Inhibitor Cocktail (Thermo Fisher Scientific, Waltham MA)) using acid-washed 1.4 mm zirconium beads and benchtop BeadBug™ tissue homogenizer (Benchmark Scientific, Sayreville, NJ). Homogenates were sonicated for 1 min in the sonication bath (Branson M1800, Branson Ultrasonics, Danbury, CT) and centrifuged at 10,000*g* for 20 min at 4 °C. Multiplexed magnetic bead-based immunoassay kit was used according to the manufacturer’s instructions. All data for cytokine/chemokine analyses are represented as the mean ± standard error. One-way ANOVA and Tukey’s post hoc test were used to determine statistical significance between genotypes with *p* < 0.05 considered significant. The 32 analyzed molecules were eotaxin (CCL11), granulocyte colony-stimulating factor (G-CSF), granulocyte-macrophage colony-stimulating factor (GM-CSF), interferon-gamma (IFN-γ), interleukin-1α (IL-1α), interleukin-1β (IL-1β), interleukin-2 (IL-2), interleukin-3 (IL-3), interleukin-4 (IL-4), interleukin-5 (IL-5), interleukin-6 (IL-6), interleukin-7 (IL-7), interleukin-9 (IL-9), interleukin-10 (IL-10), interleukin-12 (IL-12/p40), interleukin-12 (IL-12/p70), interleukin-13 (IL-13), interleukin-15 (IL-15), interleukin-17 (IL-17), interferon-gamma-induced protein 10 (IP-10/CXCL10), keratinocyte chemoattractant (KC/CXCL1), leukemia inhibitory factor (LIF), lipopolysaccharide-inducible CXC chemokine (LIX/CXCL5), monocyte chemoattractant protein-1 (MCP-1/CCL2), macrophage colony-stimulating factor (M-CSF), monokine induced by gamma interferon (MIG/CXCL9), macrophage inflammatory protein-1α (MIP-1α/CCL3), macrophage inflammatory protein-1β (MIP-1β/CCL4), macrophage inflammatory protein-2 (MIP-2/CXCL2), regulated on activation normal T cell expressed and secreted (RANTES/CCL5), tumor necrosis factor alpha (TNF-α), and vascular endothelial growth factor (VEGF). A total 45 cerebellar samples (3-week or terminal stage) from wild-type (*Npc1*^*+/+*^*/App*^*+/+*^), APP knockout (*Npc1*^*+/+*^*/App*^−/−^), NPC1 knockout (*Npc1*^−/−^*/App*^*+/+*^), NPC1 knockout/APP heterozygote (*Npc1*^−/−^/*App*^+/−^), and NPC1/APP double knockout (*Npc1*^−/−^/*App*^−/−^) mice were analyzed simultaneously. Additional file 15: Table S1 outlines the total number of mice used for the multiplex cytokine/chemokine assay.

### Immunocytochemistry

Mice brains of the indicated ages of *Npc1*^*+/+*^*/App*^*+/+*^, *Npc1*^*+/+*^*/App*^−/−^, *Npc1*^−/−^*/App*^*+/+*^, and *Npc1*^−/−^*/App*^−/−^ genotypes were processed for immunohistochemistry as described by us [6]. Sections 25 μm thick were cut sagittally through the cerebellum and mounted onto gelatin-chrome alum-coated Superfrost microscope slides (VWR, Denver, USA). Slides were placed on a warming surface at 37 °C for 30 min and rinsed with PBS for 10 min six times. Slides were incubated in blocking solution (PBS with 5% normal goat serum, 1% bovine serum albumin and 0.2% of 10% Triton x100) for 2 h at room temperature. This step was followed by a 4 °C overnight incubation with CD3 antibody at 1:200 (Abcam 135372); incubation buffer consisted of PBS with 2% normal goat serum, 1% bovine serum albumin, and 0.1% Triton X-100. Following 3 washes in PBS with 0.1% Tween-20, slides were incubated in the dark with donkey anti-rabbit 488 secondary antibody (Abcam 21206) for 2 h at room temperature; incubation buffer consisted of PBS with 2% normal goat serum, 1% bovine serum albumin and 0.1% Triton X-100. Samples were washed twice in PBS with 0.1% Tween-20 and once with PBS. Slides were mounted in Vectashield/DAPI hard-set mounting medium (Vectashield H-1500).

#### Controlled cortical impact model

We used a controlled cortical impact model as a positive control for the presence of infiltrated T lymphocytes [17, 18]. Mice were anesthetized with isoflurane (1–3%) and shaved, and the surgical area cleaned with surgical soap, isopropyl alcohol, and butadiene. A lidocaine injection was given prior to incision to expose the skull. After the skin was retracted, a 5.0-mm diameter craniectomy—centered between the bregma and lambda and 2.5 mm lateral to the sagittal suture—was performed to expose the underlying dura and cortex. The injury was induced with a 3.0-mm flat-tipped, metal impactor. The impactor was centered within the craniectomy site and impact occurred with a velocity of 5.3 m/s, depth of 1.5 mm, and dwell time of 100 ms. Immediately following injury, the injury site was cleaned of blood and a polystyrene skull-cap was placed over the craniectomy site and sealed with VetBond. The incision was sutured and mice received an injection of saline for hydration and buprenorphine for pain prevention. Mice were placed in a heated recovery chamber and monitored for 1 h prior to returning to the home cage. Daily weights were taken for the first 7 days to monitor recovery. Injury parameters resulted in a moderately severe injury composed of cortical loss without overt hippocampal loss and sustained behavioral deficits. Tissue processing and CD3+ cell evaluation was carried out on 25-μm frozen cortical sections cut between the bregma − 3.5 and 1.0 to capture the lesion.

## Results

### Genome-wide transcriptome analysis of pre-symptomatic cerebella reveals that loss of APP exacerbates the early activation of aberrant IFN-γ downstream signaling in NPC mice

Microarray hybridization yielded 39,429 transcript reads from which differentially expressed genes (DEGs) were selected by combining a fold-change cutoff (absolute change > 1.5) and a *p*-value cutoff (*p* < 0.05), as previously described [15]. From *Npc1*^*+/+*^*/App*^−/−^ cerebella, 6,269 transcript-reads (TRs) displayed an absolute fold-change (aFC) greater than 1.5 (FC < − 1.5 or FC > 1.5) and 1,534 TRs were statistically significant (*p* < 0.05) compared with the wild-type (*Npc1*^*+/+*^*/App*^*+/+*^), analyzed by one-way ANOVA and Tukey’s post hoc test (Table 1). In total, 891 DEGs were identified (following transcript ID to gene mapping), of which 418 genes were upregulated and 473 genes were downregulated. In *Npc1*^−/−^*/App*^*+/+*^ samples, 3,967 TRs displayed aFC > 1.5 and 684 TRs were statistically significant (*p* < 0.05). In total, 431 DEGs were identified (following transcript ID to gene mapping), of which 252 genes were upregulated and 179 genes were downregulated (Table 1). In *Npc1*^−/−^*/App*^−/−^ cerebella, 7,132 TRs displayed aFC > 1.5 and 3,359 TRs were statistically significant (*p* < 0.05). In total, 1,973 DEGs were identified (following transcript ID to gene mapping), of which 1,265 genes were upregulated and 708 genes were downregulated (Table 1). The overall transcript expression of the *Npc1*^*+/+*^*/App*^−/−^ and *Npc1*^−/−^*/App*^−/−^ datasets are illustrated in Additional file 1: Figure S1, Additional file 2: Figure S2 and Additional file 3: Figure S3.

**Table 1:** Differentially expressed genes identified in each genotype by genome-wide transcriptome analysis. *TR* number of transcript-reads by microarray, *aFC* absolute fold-change, *DEG* differentially expressed gene (mapped ID + statistically significant by FC and *p* cutoffs)

	*Npc1*^*+/+*^*/App*^−/−^	*Npc1*^−/−^*/App*^*+/+*^	*Npc1*^−/−^*/App*^−/−^
TR (aFC > 1.5)	6269	3967	7132
TR (*p* < 0.05)	1534	684	3359
DEG (FC + *p*)	891	431	1973
DEG (up)	418	252	1265
DEG (down)	473	179	708

**Fig. 1:**
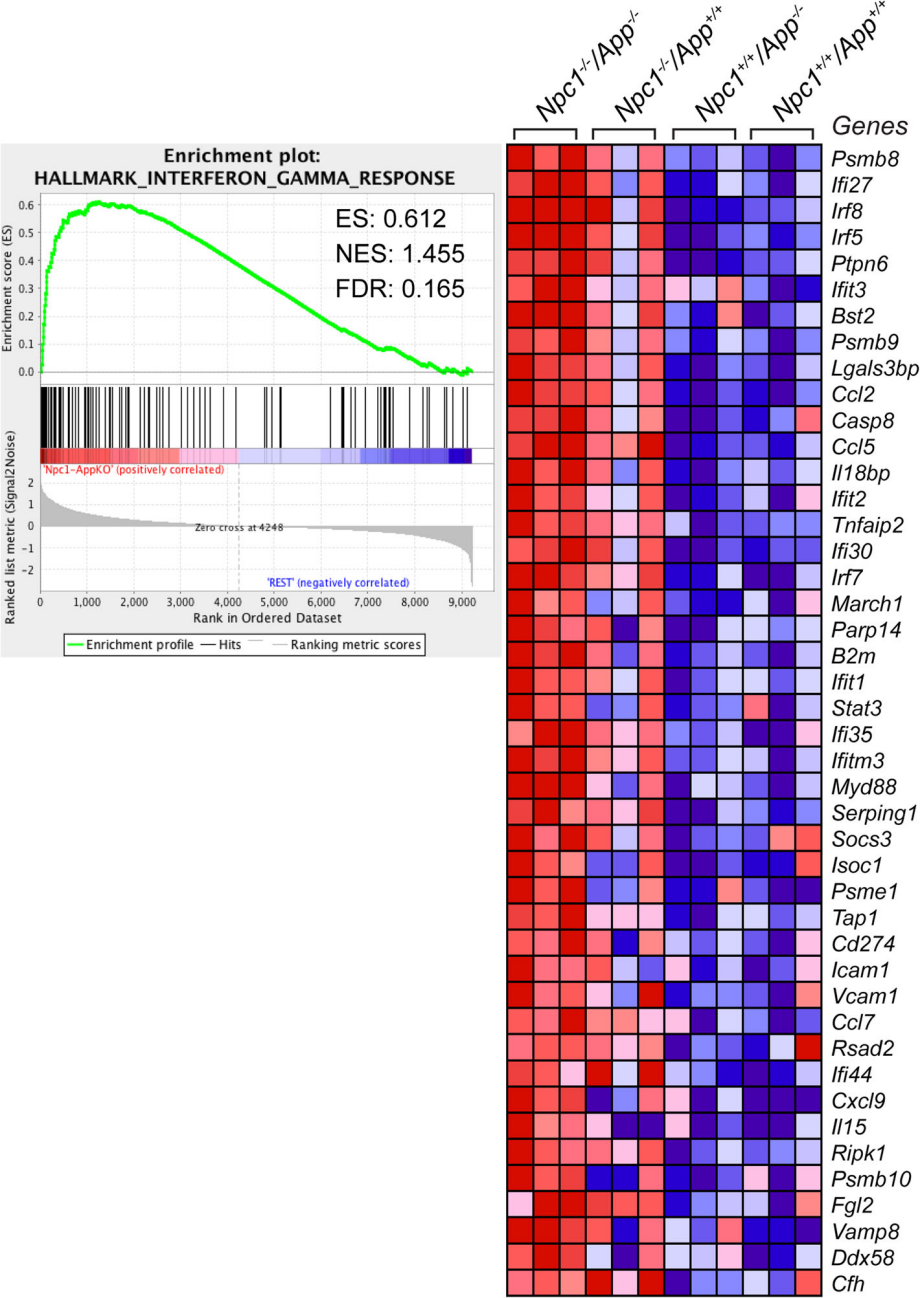
Activation of IFN-γ signaling gene set in the *Npc1*^−/−^*/App*^−/−^ mouse cerebella. GSEA reveals the activation of *Interferon Gamma Response* gene sets in *Npc1*^−/−^*/App*^−/−^ mouse cerebella compared with the three remaining genotypes (Npc1^−/−^/App^−/−^ vs. remaining genotypes). ES = enrichment score, NES = normalized enrichment score, FDR-*q* = false discovery rate *q* value

**Fig. 2:**
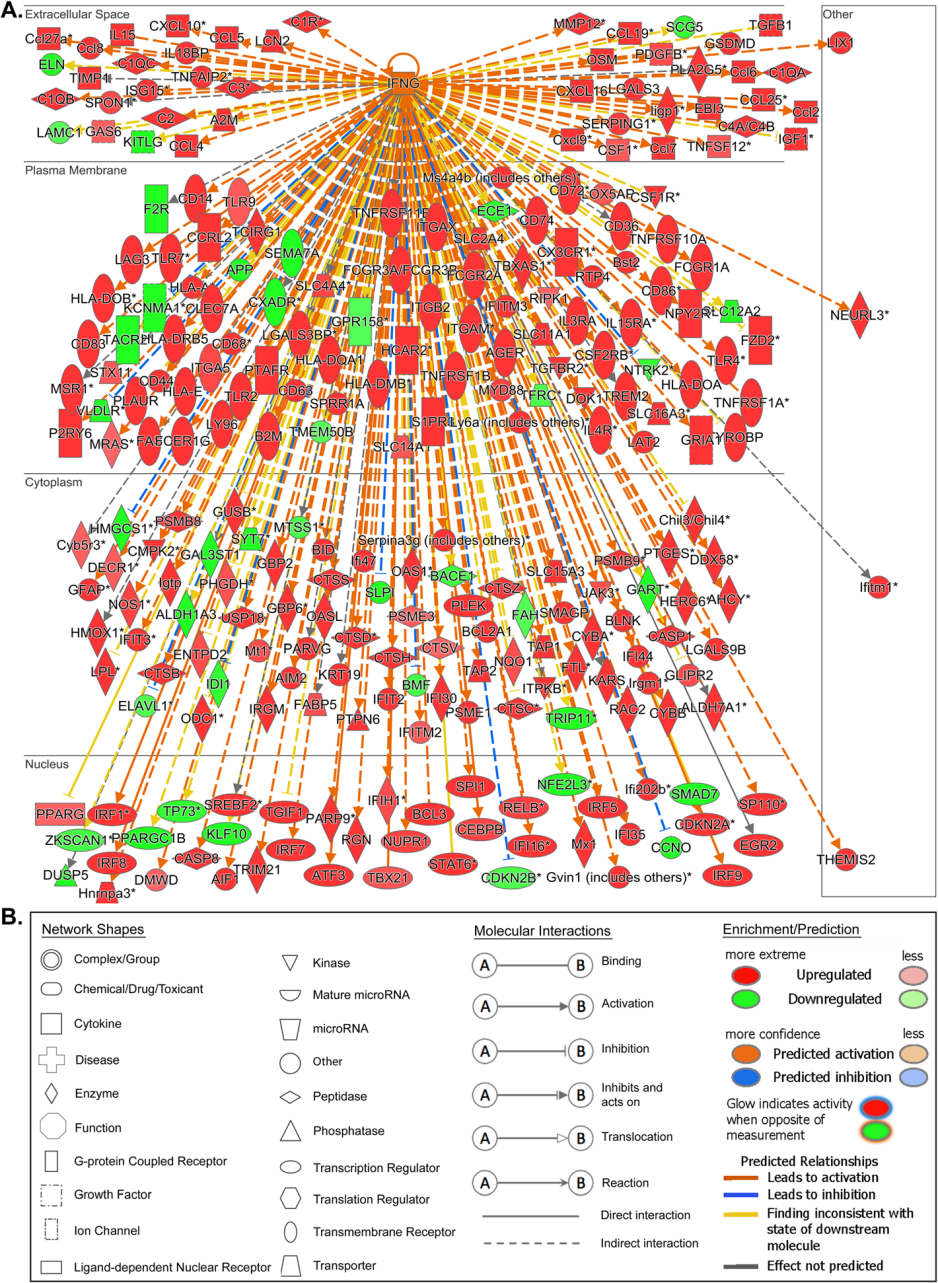
Robust activation of IFN-γ-responsive genes in the *Npc1*^−/−^*/App*^−/−^ mouse cerebella. **a** Two hundred and sixty-two IFN-γ-responsive genes are differentially expressed in the *Npc1*^−/−^*/App*^−/−^ cerebella compared with age-matched wild-type littermates (*Npc1*^*+/+*^*/App*^*+/+*^). All differentially expressed genes (DEGs) are localized to their sub-cellular location. All plotted DEGs meet the significance cutoff of fold-change (absolute FC > 1.5) and *p*-value (*p* < 0.05). *Duplicate identifiers used for the same gene. **b** IPA key for molecule shape, color, and interaction

Comparative analyses of wild-type cerebella vs. *Npc1*^*+/+*^*/App*^−/−^, *Npc1*^−/−^*/App*^*+/+*^, and *Npc1*^−/−^*/App*^−/−^ revealed that the loss of APP results in a significant exacerbation of the aberrant IFN-γ downstream signaling previously characterized in pre-symptomatic *Npc1*^−/−^*/App*^*+/+*^ mice [5]. Gene set enrichment analysis (GSEA) revealed that *Interferon Gamma Signaling* gene set was significantly enriched in the *Npc1*^−/−^*/App*^−/−^ mouse cerebellar transcriptome (NES = 1.455 and FDR = 0.165), in comparison to *Npc1*^*+/+*^*/App*^*+/+*^, *Npc1*^*+/+*^*/App*^−/−^, and *Npc1*^−/−^*/App*^*+/+*^ (Fig. 1). Ingenuity Pathway Analysis confirmed that *Npc1*^−/−^*/App*^−/−^ mouse cerebellar transcriptome indeed displayed a significant increase in IFN-γ-responsive genes (Fig. 2). Compared with a single knockout mouse model of NPC (*Npc1*^−/−^*/App*^*+/+*^) which displayed aberrant differential expression of 60 IFN-γ-responsive genes in the pre-symptomatic stage, *Npc1*^−/−^*/App*^−/−^ mouse cerebella displayed the differential expression of 262 IFN-γ-responsive genes (Fig. 2). Of those, 223 were upregulated and 39 were downregulated. In addition, IPA *Upstream Analysis* revealed that IFN-γ is the most likely upstream master regulator of 1,973 DEGs identified in the *Npc1*^−/−^*/App*^−/−^ mouse cerebellar transcriptome (Table 2). This finding is congruent with our previous report that IFN-γ is the top master regulator of 387 DEGs identified in the *Npc1*^−/−^ cerebellar transcriptome [5].

**Table 2:**
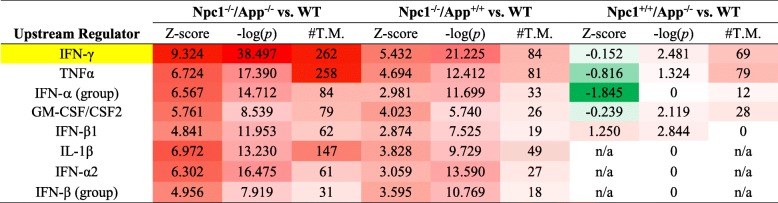
Top 8 predicted cytokine/chemokine upstream regulators of DEGs identified in *Npc1*^−/−^*/App*^−/−^, *Npc1*^−/−^*/App*^*+/+*^, and *Npc1*^*+/+*^*/App*^−/−^ mouse cerebella. IPA *Upstream Analysis* and *Comparison Analysis* identified eight cytokines and chemokines upstream master regulators in each genotype, compared with the wild-type (*Npc1*^*+/+*^*/App*^*+/+*^) littermates. Each of the three columns (*Z*-score, − log(*p*), and #T.M.) across the three genotypes are heatmaps. Red = enriched, green = down, and white = zero*. Z*-scores and *p* values calculated by IPA software. *#T.M.* number of downstream target molecules, *WT* wild type

### Loss of APP exacerbates the early activation of aberrant IFN-**γ** downstream signaling in NPC mice

**Fig. 3:**
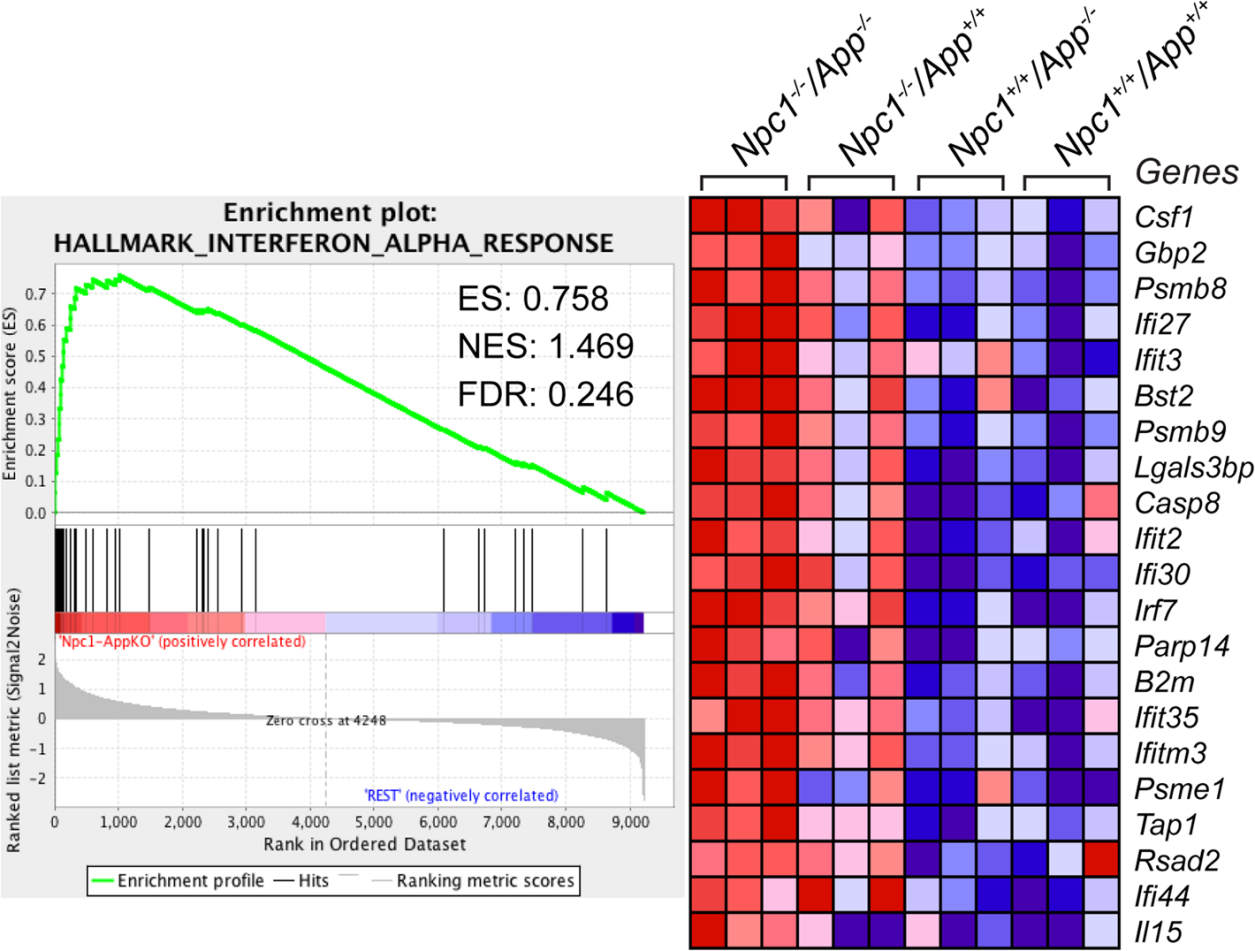
Activation of IFN-α signaling gene set in the *Npc1*^−/−^*/App*^−/−^ mouse cerebella. GSEA reveals the activation of Interferon Alpha Response gene sets in *Npc1*^−/−^*/App*^−/−^ mouse cerebella compared with the three remaining genotypes (*Npc1*^−/−^*/App*^−/−^ vs. remaining genotypes). ES = enrichment score, NES = normalized enrichment score, FDR-*q* = false discovery rate *q* value

**Fig. 4:**
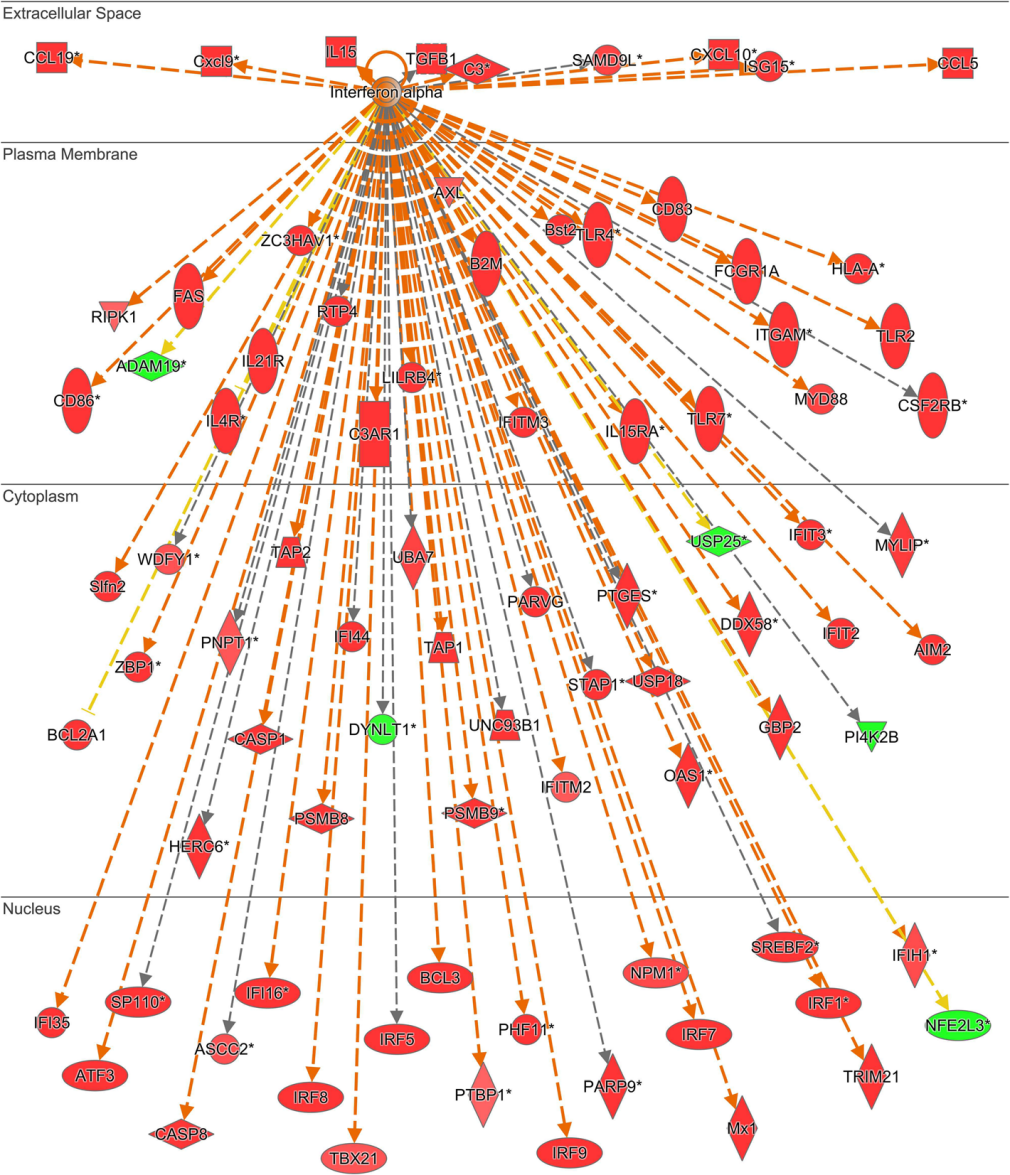
Robust activation of IFN-α-responsive genes in the *Npc1*^−/−^*/App*^−/−^ mouse cerebella. Eighty-four IFN-α-responsive genes are differentially expressed in the *Npc1*^−/−^*/App*^−/−^ cerebella compared with age-matched wild-type littermates (*Npc1*^*+/+*^*/App*^*+/+*^). All differentially expressed genes (DEGs) are localized to their sub-cellular location. All plotted DEGs meet the significance cutoff of fold-change (absolute FC > 1.5) and *p*-value (*p* < 0.05). *Duplicate identifiers used for the same gene. A detailed key for IPA molecular shape, color, and interaction is provided in Fig. 2

Comparative analyses of wild-type cerebella versus *Npc1*^*+/+*^*/App*^−/−^, *Npc1*^−/−^*/App*^*+/+*^, and *Npc1*^−/−^*/App*^−/−^ also revealed that loss of APP exacerbates the aberrant IFN-α downstream signaling seen in pre-symptomatic *Npc1*^−/−^*/App*^*+/+*^ mice [5]. GSEA showed that the *Interferon Alpha Signaling* gene set is significantly enriched in the *Npc1*^−/−^*/App*^−/−^ mouse cerebellar transcriptome (NES = 1.469 and FDR = 0.246), when compared with the *Npc1*^*+/+*^*/App*^*+/+*^, *Npc1*^*+/+*^*/App*^−/−^, and *Npc1*^−/−^*/App*^*+/+*^ genotypes (Fig. 3). IPA further confirmed that 84 IFN-α-responsive genes are differentially expressed in *Npc1*^−/−^*/App*^−/−^ mouse cerebella when compared with wild-type (*Npc1*^*+/+*^*/App*^*+/+*^) controls (Fig. 4). Of the 84 DEGs, 79 IFN-α-responsive genes were upregulated and 5 IFN-α-responsive genes were downregulated (Fig. 4). This is a substantial increase from the differential expression of 23 IFN-α-responsive genes in *Npc1*^−/−^ mice versus wild-type controls [5].

### Loss of APP results in the exacerbation of NPC-specific inflammatory pathways mediated by IFN-γ- and IFN-α-responsive genes

Our previous work showed that four major inflammatory pathways are aberrantly activated in pre-symptomatic *Npc1*^−/−^ mouse cerebella: activation of microglia, anti-viral response, and T-lymphocyte activation and chemotaxis [5]. Here, we found that, in *Npc1*^−/−^*/App*^−/−^ mice, the aberrant activation of all four NPC-specific inflammatory pathways was exacerbated: IPA *Disease and Function Analysis* revealed strong activation of microglia in the *Npc1*^−/−^*/App*^−/−^ mouse cerebellum, as measured by the identification of 29 significant DEGs associated with this pathway (Additional file 4: Figure S4). Of these, 25 were IFN-γ-responsive genes and 7 were IFN-α-responsive, a substantial change from the 9 IFN-γ-responsive and 5 IFN-α-responsive genes related to microglial activation previously identified in the *Npc1*^−/−^ cerebellum [5]. Antiviral response was also strongly activated in *Npc1*^−/−^*/App*^−/−^ mouse cerebella, as revealed by the presence of 56 DEGs related to this pathway (Additional file 5: Figure S5), 47 of which were IFN-γ-responsive and 39 IFN-α-responsive, again representing a substantial increase compared with the 15 IFN-γ-responsive and 9 IFN-α-responsive altered genes previously identified in the *Npc1*^−/−^ cerebellum [5]. *Disease and Function Analysis* and *Upstream Analysis* also identified 83 significantly DEGs related to antimicrobial response in the *Npc1*^−/−^*/App*^−/−^ cerebella transcriptome compared with wild-type mice (*Npc1*^*+/+*^*/App*^*+/+*^; Additional file 6: Figure S6). Of those, 62 were IFN-γ-responsive genes and 44 were IFN-α-responsive genes. The DEGs involved in activation of antimicrobial response showed a significant overlap (56 genes) with the antiviral response (Additional file 5: Figure S5) but additional genes involved in antimicrobial immune response were also identified (Additional file 6: Figure S6).

Activation of T lymphocytes was also present in *Npc1*^−/−^*/App*^−/−^ cerebella, as evidenced by the presence of 87 linked DEGs (Additional file 7: Figure S7). Of these, 77 were IFN-γ-responsive and 34 were IFN-α-responsive. Interestingly, IPA also showed that T-lymphocyte co-stimulatory ligand receptor CD28 was also implicated in the *Npc1*^−/−^*/App*^−/−^ cerebellum (Additional file 8: Figure S8). CD28 is a T-lymphocyte co-receptor for membrane-bound-ligands on antigen-presenting cells, such as CD80 and CD86, that are required for T-lymphocyte activation and survival [19]. In *Npc1*^−/−^*/App*^−/−^ cerebella, 42 DEGs downstream of predicted CD28 activation were identified by IPA (Additional file 8: Figure S8), thereby providing additional insight into the potential mechanism by which APP loss of function may contribute to the IFN-mediated T-lymphocyte activation seen in the *Npc1*^−/−^*/App*^−/−^ mouse cerebella (Additional file 7: Figure S7). IPA also showed that the aberrant expression of DEGs related to chemotaxis of T-lymphocytes in NPC is exacerbated by the loss of APP (Additional file 9: Figure S9). In *Npc1*^−/−^*/App*^−/−^, 25 DEGs related to chemotaxis of T-lymphocytes were identified, of which 18 were IFN-γ-responsive and 8 were IFN-α-responsive (Additional file 9: Figure S9). By comparison, we had previously identified 6 IFN-γ-responsive genes and 4 IFN-α-responsive genes related to chemotaxis of T-lymphocytes in *Npc1*^−/−^ cerebella [5].

IPA *Disease and Function Analysis* identified 87 significantly DEGs related to the activation of antigen-presenting cells (APCs) in *Npc1*^−/−^*/App*^−/−^ mice, compared with wild-type controls (*Npc1*^*+/+*^*/App*^*+/+*^; Additional file 10: Figure S10). The combination of IPA *Disease and Function Analysis* and *Upstream Analysis* further identified 85 IFN-γ-responsive genes and 35 IFN-α-responsive genes related to antigen presentation in *Npc1*^−/−^*/App*^−/−^ cerebella, highlighting antigen presentation as one of the main inflammatory mechanisms related to APP loss of function in the NPC brain (Additional file 10: Figure S10). More specifically, the activation of dendritic cells was implicated in *Npc1*^−/−^*/App*^−/−^ mouse cerebella, as IPA *Disease and Function Analysis* unveiled 27 DEGs linked to this pathway (Additional file 11: Figure S11). Of these, 25 were IFN-γ-responsive and 17 were IFN-α-responsive, further validating the notion of IFN exacerbation as a consequence of APP loss of function. Finally, IPA *Upstream Analysis* showed that genes downstream of the co-stimulatory molecules involved in APC-mediated activation of the adaptive immune system are significantly enriched in *Npc1*^−/−^*/App*^−/−^ cerebella, with 32 DEGs mapping to CD40, 6 mapping to CD86, and 4 mapping to ICAM1 (Additional file 12: Figure S12).

### Multiplex protein analysis across *Npc1* and *App* genotypes: NPC pre-symptomatic stage

**Fig. 5:**
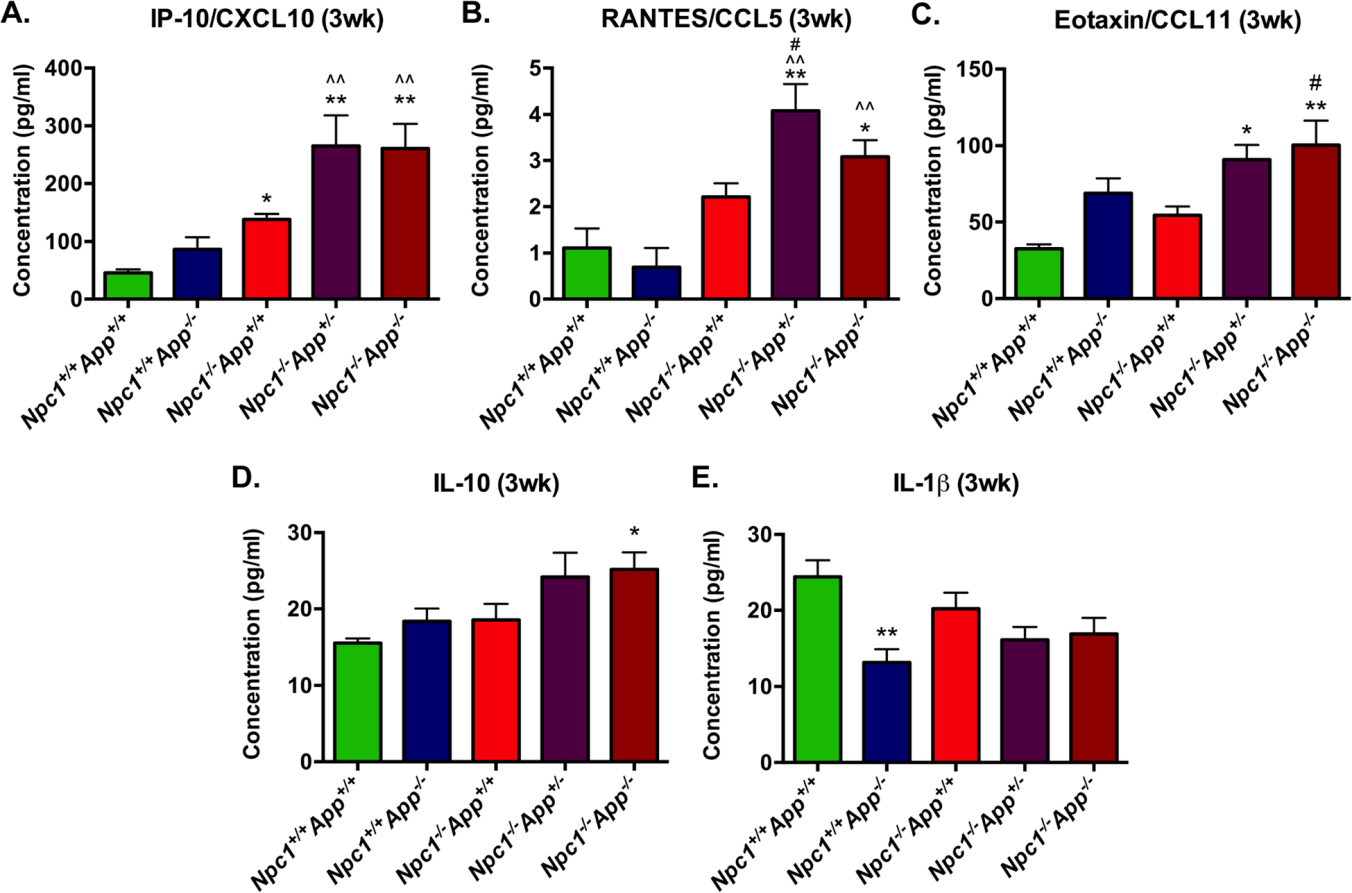
Differentially expressed cytokines and chemokines detected by multiplex protein analysis in the 3-week old mouse cerebella across five genotypes. Progressive loss of functional *App* allele in NPC mouse model (*Npc1*^−/−^*/App*^+/−^ and *Npc1*^−/−^*/App*^−/−^) resulted in significant increase of pro-inflammatory cytokines at 3 weeks of age. Cytokines were measured by multiplexed magnetic bead-based immunoassay kit (Catalog# MCYTMAG-70 K-PX32, Millipore Sigma, Burlington MA). **a** IFN-γ-responsive cytokine IP-10/CXCL10 is the only protein significantly increased in *Npc1*^−/−^*/App*^*+/+*^ in the pre-symptomatic mouse cerebella. This increased expression is significantly exacerbated with the loss of APP function (compare *Npc1*^−/−^*/App*^*+/+*^ with *Npc1*^−/−^*/App*^+/−^ and *Npc1*^−/−^*/App*^−/−^). **b**–**d** RANTES/CCL5, EOTAXIN/CCL11, and IL-10 were also significantly increased in *Npc1*^−/−^*/App*^+/−^ and/or *Npc1*^−/−^*/App*^−/−^ mouse cerebella compared with wild-type (*Npc1*^*+/+*^*/App*^*+/+*^) and/or *Npc1*^−/−^*/App*^*+/+*^. **e** IL-1β expression was reduced in mice lacking APP function (*Npc1*^*+/+*^*/App*^−/−^). Values are means ± SEM. *p < 0.05, **p < 0.01. * = compared to *Npc1*^*+/+*^*/App*^*+/+*^; ^ = compared with *Npc1*^*+/+*^*/App*^−/−^; # = compared with *Npc1*^−/−^*/App*^*+/+*^

In order to identify how the loss of each *App* allele affects the protein expression of pro- and anti-inflammatory cytokines (downstream of IFN signaling), we utilized a multiplex cytokine analysis to simultaneously determine the protein levels of 32 cytokines in the following genotypes: *Npc1*^*+/+*^*/App*^*+/+*^, *Npc1*^*+/+*^*/App*^−/−^, *Npc1*^−/−^*/App*^*+/+*^, *Npc1*^−/−^*/App*^+/−^, and *Npc1*^−/−^*/App*^−/−^. In 3-week-old cerebella across all five genotypes, 26 cytokines were expressed within detectable levels but only 5 of them displayed significant differential expression in either *Npc1*^−/−^*/App*^*+/+*^, *Npc1*^−/−^*/App*^+/−^, or *Npc1*^−/−^*/App*^−/−^ compared with wild-type littermate control (Fig. 5). IFN-γ downstream effector cytokine, IP-10/CXCL10, was the only cytokine significantly increased in *Npc1*^−/−^*/App*^*+/+*^ at 3 weeks (Fig. 5a [5];), and loss of a single *App* allele in the Npc1 brain (*Npc1*^−/−^*/App*^+/−^) was sufficient to trigger an additional increase in IP-10/CXCL10 expression.

In addition, one IFN-γ downstream cytokine, RANTES/CCL5, displayed an increased trend in 3-week-old *Npc1*^−/−^*/App*^*+/+*^mice compared with wild-type littermates, but did not reach statistical significance (Fig. 5b). By contrast, loss of a single *App* allele in NPC mice (*Npc1*^−/−^*/App*^+/−^) was sufficient to significantly increase its expression (Fig. 5b).

Eotaxin/CCL11 was increased in *Npc1*^−/−^*/App*^*+/+*^, but this increase did not reach statistical significance (Fig. 5c). Interestingly, loss of *App* in a wild-type background also showed an increased trend, but the impact of *App* loss on eotaxin expression is only significant in the NPC brain following loss of both *App* alleles (Fig. 5c). Expression of IL-10 was not significantly altered in single gene knockouts (*Npc1*^*+/+*^*/App*^−/−^ and *Npc1*^−/−^*/App*^*+/+*^) at 3 weeks of age, compared with wild-type controls (Fig. 5d). However, in the NPC brain, loss of both *App* alleles (*Npc1*^−/−^*/App*^−/−^) resulted in a statistically significant increase in expression (Figure 5d). Lastly, IL-1β displayed a trend toward a decrease in *Npc1*^−/−^*/App*^*+/+*^ (Fig. 5e), which did not reach statistical significance. However, loss of App in a wild-type Npc1 background (*Npc1*^*+/+*^*/App*^−/−^) led to a significant decrease in expression (Fig. 5e). Interestingly, loss of App in the NPC brain also tended to decrease IL-1b expression, but such reduction did not reach statistical significance.

### Multiplex protein analysis across *Npc1* and *App* genotypes: NPC post-symptomatic stage

Next, we measured the levels of the 32 prominent pro- and anti-inflammatory cytokines described above in the terminal stage cerebella of mice across the same five genotypes: *Npc1*^*+/+*^*/App*^*+/+*^, *Npc1*^*+/+*^*/App*^−/−^, *Npc1*^−/−^*/App*^*+/+*^, *Npc1*^−/−^*/App*^+/−^, and *Npc1*^−/−^*/App*^−/−^. The average age of the humane endpoint of this animal study were: 11.1 weeks for *Npc1*^−/−^*/App*^*+/+*^, 10.4 weeks for *Npc1*^−/−^*/App*^+/−^, and 9.4 weeks for *Npc1*^−/−^*/App*^−/−^. *Npc1*^*+/+*^*/App*^*+/+*^ and *Npc1*^*+/+*^*/App*^−/−^ littermates were assessed at 12 weeks of age. In total, 26 cytokines/chemokines were detected in the terminal stage or 12-week cerebella and 20 displayed significant differential expression in either *Npc1*^−/−^*/App*^*+/+*^, *Npc1*^−/−^*/App*^+/−^, or *Npc1*^−/−^*/App*^−/−^ (Additional file 13: Figure S13). Levels of six cytokines/chemokines were below the detectable range of the assay (GM-CSF, IL-3, IL-12(p70), IL-13, LIX/CXCL5, and TNFα; data not shown).

For comparative expression analysis, wild-type (*Npc1*^*+/+*^*/App*^*+/+*^) and *App* gene knockout (*Npc1*^*+/+*^*/App*^−/−^) mice were used as primary and secondary controls, respectively. In total, seven cytokines/chemokines were increased in the terminal stage NPC (*Npc1*^−/−^*/App*^*+/+*^) cerebella (Additional file 13: Figure S13A-G). Of the seven cytokines/chemokines that showed significant increase in *Npc1*^−/−^*/App*^*+/+*^ mutants compared with wild-type controls (*Npc1*^*+/+*^*/App*^*+/+*^), two (IL-1α and MIP-1α/CCL4) were also increased in *Npc1*^−/−^*/App*^+/−^ and *Npc1*^−/−^*/App*^−/−^ (Additional file 13: Figure S13A & B) and two (KC/CXCL5 and LIF) showed a non-significant increase in *Npc1*^−/−^*/App*^+/−^ that reached significance with the loss of both App alleles (*Npc1*^−/−^*/App*^−/−^) (Additional file 13: Figure S13C & D). IP-10/CXCL10 and EOTAXIN/CCL11 showed significant increase in the terminal stage *Npc1*^−/−^*/App*^*+/+*^ mouse cerebella compared with wild-type controls (*Npc1*^*+/+*^*/App*^*+/+*^), but did not increase in either *Npc1*^−/−^*/App*^+/−^ or *Npc1*^−/−^*/App*^−/−^ samples (Additional file 13: Figure S13E & F). RANTES/CCL5 showed a significant increase in the terminal stage *Npc1*^−/−^*/App*^*+/+*^ mouse cerebella compared with wild-type controls (*Npc1*^*+/+*^*/App*^*+/+*^), an effect counteracted by *App* loss (Additional file 13: Figure S13G).

Two cytokines/chemokines (IL-12(p40) and IL-15) showed a significant decrease in the terminal stage *Npc1*^−/−^*/App*^*+/+*^ mouse cerebella compared with wild-type controls (*Npc1*^*+/+*^*/App*^*+/+*^), and their levels were further decreased in *Npc1*^−/−^*/App*^+/−^ and/or *Npc1*^−/−^*/App*^−/−^ (Additional file 13: Figure S13H & I). Lastly, five cytokines/chemokines (IL-5, IL-7, G-CSF, IFN-γ, and IL-1β) showed no changes in the terminal stage *Npc1*^−/−^*/App*^*+/+*^ mouse cerebella compared with wild-type controls (*Npc1*^*+/+*^*/App*^*+/+*^), but their levels were decreased in *Npc1*^−/−^*/App*^+/−^ and/or *Npc1*^−/−^*/App*^−/−^ samples (Additional file 13: Figure S13 J-N). Altogether, it is interesting to note that cytokine/chemokine expression levels in terminal stage *Npc1*^−/−^*/App*^+/−^ or *Npc1*^−/−^*/App*^−/−^ mouse cerebella were relatively lower than those of *Npc1*^−/−^*/App*^*+/+*^ (Additional file 13: Figure S13). MIP-1β/CCL4 was the only exception to this general pattern (Additional file 13: Figure S13B).

### T cell infiltration across *Npc1* and *App* genotypes

**Fig. 6:**
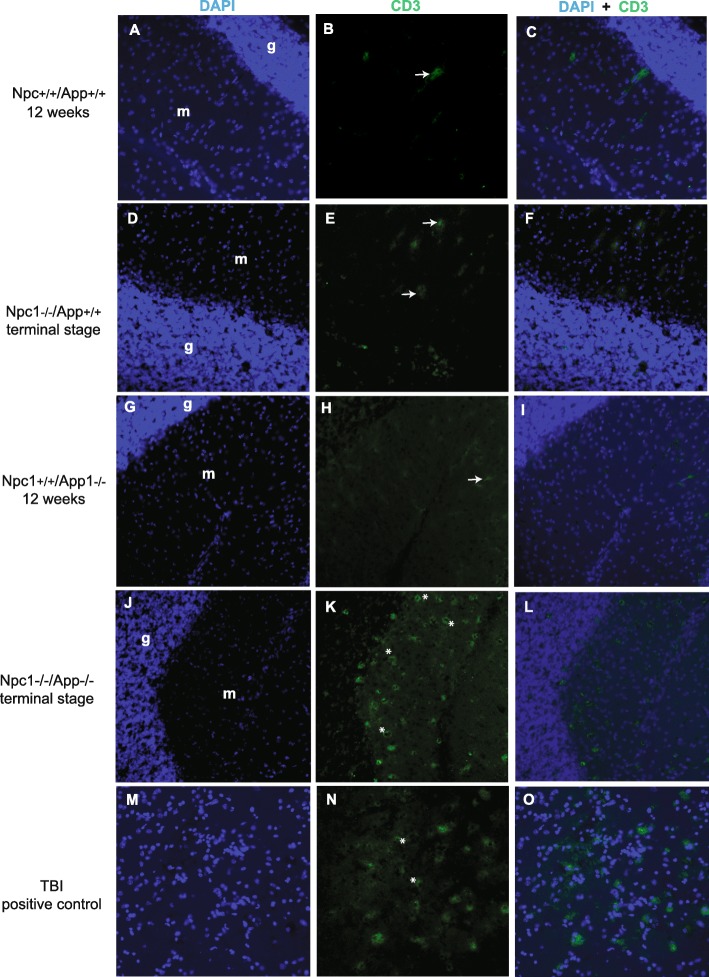
Infiltration of CD3+ T cells in cerebellum. Immunohistochemical staining reveals the presence of CD3+ cells in the molecular layer of the cerebellum of *App*^−/−^*/Npc1*^−/−^ mice at terminal disease stage, but not in any other genotypes. Shown for comparison as a positive control is CD3 staining of T cells in mice following a traumatic brain injury protocol as described in the Methods section. **a**–**c** *Npc1*^*+/+*^*/App*^*+/+*^ mice at 12 weeks of age. **d**–**f** *Npc1*^−/−^*/App*^*+/+*^ mice at terminal disease stage. **g**–**i** *Npc1*^*+/+*^*/App*^−/−^ mice at 12 weeks of age. **j–l** *App*^−/−^*/Npc1*^−/−^ mice at terminal disease stage. g: granular layer of the cerebellum; m: molecular layer of the cerebellum. **m**–**o** Traumatic brain injury positive control. Shown is the lesion area, as described in the Methods section. White asterisks show CD3+ cells and white arrows show areas of stained patterns artifactual in nature, as they appear in all genotypes and all ages tested

Because of the predicted effect of IP-10 increased expression on T cell activation and chemotaxis [20, 21], we measured T cell infiltration across *Npc1*^*+/+*^*/App*^*+/+*^, *Npc1*^*+/+*^*/App*^−/−^, *Npc1*^−/−^*/App*^*+/+*^, and *Npc1*^−/−^*/App*^−/−^ genotypes, at three weeks of age as well as 12 weeks (*Npc1*^*+/+*^*/App*^*+/+*^, *Npc1*^*+/+*^*/App*^−/−^) or humane endpoint terminal stage (*Npc1*^−/−^*/App*^*+/+*^ and *Npc1*^−/−^*/App*^−/−^). As shown in Fig. 6, T cell infiltration was indeed evident in *Npc1*^−/−^*/App*^−/−^ cerebellum at the terminal stage (Fig. 6 h). No evidence of T cell infiltration was found in 3-week old mice of any Npc1 or App genotypes (Additional file 14: Figure S14).

## Discussion

Our comparative and systematic genome-wide transcriptome analyses of *Npc1*^*+/+*^*/App*^*+/+*^, *Npc1*^*+/+*^*/App*^−/−^, *Npc1*^−/−^*/App*^*+/+*^, and *Npc1*^−/−^*/App*^−/−^ mice at pre-symptomatic stage revealed that loss of APP function results in severe exacerbation of multiple inflammatory pathways already present in the NPC brain. Specifically, GSEA and IPA *Upstream Analysis* showed significantly increased expression of IFN-γ- and IFN-α-responsive genes in the *Npc1*^−/−^*/App*^−/−^ cerebellar transcriptome (Figs. 1, 2, 3, and 4; 262 IFN-γ-responsive and 84 IFN-α-responsive genes; Figs. 2 and 4), when compared with *Npc1*^−/−^*/App*^*+/+*^ mice (60 IFN-γ-responsive and 23 IFN-γ-responsive genes [5];), consistent with the significant exacerbation of all four major inflammatory pathways previously identified in this mouse model of NPC [5], namely activation of microglia, anti-viral response, activation of T-lymphocytes, and chemotaxis of T-lymphocytes (Additional file 4: Figure S4, Additional file 5: Figure S5, Additional file 7: Figure S7 and Additional file 9: Figure S9).

The mechanisms by which APP loss may cause an exacerbation of inflammatory pathways prior to disease onset in NPC is not immediately clear. APP processing is abnormal in the NPC brain, as evidenced by an increase in amyloid peptide Aβ expression, possibly due to the formation of aberrantly enlarged endosomes, a necessary compartment for the generation of Aβ [6]. Thus, it would appear reasonable to link excess Aβ expression in the NPC with its pathogenesis. However, loss of APP and, by extension, of Aβ, in the NPC brain, leads to decreased life span, increased cholesterol abnormalities and, notably, disruption of tau homeostasis [6], as well as an early exacerbation of inflammation, as shown here. These findings suggest that Aβ expression is not a primary pathogenic factor in NPC. Rather, given that APP is a multi-potent cytoprotective molecule, whose cleaved products provide beneficial effects against oxidative stress, metabolic stress, and pathogenic infections, it seems more likely that APP plays a homeostatic role in the brain and that loss of that role accelerates NPC onset and progression. For example, both monomeric and oligomeric forms of Aβ have been characterized to possess potent anti-oxidant activity [7, 8] and the function of APP intracellular domain (AICD) as a transcription factor has recently been shown to directly regulate the cytoprotective mechanisms against oxysterol-mediated stress [9]. Furthermore, Aβ has potent anti-microbial activity against many strains of pathogens, including bacteria, viruses, and yeast [10–13].

Overall, the available evidence suggests that loss of APP function in the *Npc1*^−/−^*/App*^−/−^ brain may contribute to the early altered expression of genes directly related to immune response pathways against pathogens, including *Antimicrobial Response* and *Antiviral Response* identified by IPA analysis (Additional file 5: Figure S5 and Additional file 6: Figure S6). Interestingly, compared with the sole activation of *Antiviral Response* identified by IPA in pre-symptomatic NPC, APP loss resulted in an additional enrichment of the larger functional *Antimicrobial Response*, which included 31 additional antimicrobial genes (Additional file 6: Figure S6). This increase in anti-microbial function is further highlighted by the activation of genes involved in T-lymphocyte activation and chemotaxis, as well as the activation of antigen presenting cells, all of which are crucial in host-immune response against various strains of pathogens (Additional file 7: Figure S7, Additional file 8: Figure S8, Additional file 9: Figure S9 and Additional file 10: Figure S10).

It is also noteworthy that changes in gene expression in pre-symptomatic NPC as a result of *App* deletion (*Npc1*^−/−^*/App*^−/−^) translated into increased expression of pro-inflammatory cytokines and chemokines (Fig. 5), even with the loss of one single *App* allele. This was the case with the protein expression of IP-10/CXCL10, the central downstream effector of IFN-γ identified in pre-symptomatic *Npc1*^−/−^ mice (Fig. 5a [5];), as well as several other cytokines, including RANTES, eotaxin/CCL11 and IL-10 (Fig. 5). Interestingly, the notion that haploinsufficiency of *APP* is a risk factor for neurotoxicity has been proposed in a model of copper-mediated CNS cytotoxicity [20]. In that study, a single allele loss of *App* in mice was sufficient to alter copper homeostasis comparable to that of mice lacking both alleles of *App* [20]. Therefore, it is plausible that dysregulation of APP function may exacerbate the inflammatory response and poor prognosis of NPC in humans.

Functionally, IP-10/CXCL10 is a potent downstream effector of IFN-γ [21, 22], the master regulator of the adaptive immune activation that is crucial in the transition from the innate immune response to the antigen-specific adaptive immune response [23]. IP-10/CXCL10 binds to CXCR3, on activated immune cells such as activated T-lymphocytes or natural killer cells to drive the chemotaxis of CXCR3+ cells to the site of inflammation [21, 24]. Furthermore, IP-10/CXCL10 also plays a major role in the development and antigen-specific activation of T-lymphocytes [21]. In addition, interferon-inducible T cell alpha chemoattractant (I-TAC/CXCL11) also binds the same CXCR3 receptor to elicit similar physiological functions [25–28]. The fact that T cell infiltration is apparent in the *Npc1*^−/−^/*App*^−/−^ cerebellum (Fig. 6) supports the notion that APP loss may exert its deleterious effect through IP-10/CXCL10-driven T-lymphocyte activation and chemotaxis.

In both *Npc1*^−/−^/*App*^+/−^ and *Npc1*^−/−^/*App*^−/−^ mouse cerebella, another major cytokine significantly increased at 3 weeks of age was eotaxin/CCL11 (Fig. 5c). Eotaxin/CCL11 is a potent eosinophil chemoattractant, implicated in various eosinophil-related pathogenic processes such as asthma and airway inflammation [29]. While the combined functional roles of eosinophils and eotaxin/CCL11 are widely characterized in the periphery, the precise role of both in the CNS is not well defined [30]. For example, eotaxin/CCL11 is an anti-inflammatory Th2 cytokine in the CNS in a murine model of multiple sclerosis [31]. On the other hand, astrocyte-mediated release of eotaxin/CCL11 and subsequent enhancement of neuronal death *via* increased production of microglial reactive oxygen species have also been reported [30]. In the context of the early and widespread activation of IFN-γ-responsive signaling that occurs in pre-symptomatic NPC brains [5], IFN-γ potentiates the subsequent release of eotaxin/CCL11 in the periphery [29], thereby suggesting a potential for the co-activation of IFN-γ and eotaxin/CCL11 under certain inflammatory conditions. Interestingly, co-expression of IP-10/CXCL10 receptor CXCR3 and eotaxin/CCL11 receptor CCR5 (whose ligands also include MIP-1α/CCL3, MIP-1β/CCL4, and RANTES/CCL5) have been characterized in autoimmune T-lymphocytes [32], consistent with the co-activation of CXCR3 and CCR5 as a potential pathologic mechanism involved in autoimmunity.

Loss of APP also showed a significant impact on the expression pattern of cytokines and chemokines in terminal-stage brains, as illustrated in Additional file 13: Figure S13. Interestingly, the overall expression of pro-inflammatory cytokines and chemokines in the terminal stage *Npc1*^−/−^*/App*^+/−^ or *Npc1*^−/−^*/App*^−/−^ were relatively lower than that of *Npc1*^−/−^*/App*^*+/+*^ (Additional file 13: Figure S13). While the precise mechanism responsible for this phenomenon remains to be elucidated, one plausible explanation is the significant reduction in brain mass and paralleled neuronal death observed in the *Npc1*^−/−^*/App*^−/−^ terminal stage cerebella [6]. Contrary to the classical understanding of neuronal secretion of cytokines, recent evidence consistently highlights neurons as a major source of proinflammatory cytokines and chemokines under various cytotoxic stresses within the CNS [33–35]. The difference in age-at-collection may be another confounding factor for the terminal stage cytokine/chemokine expressions, as the average age for humane-endpoint varied by a week with the successive loss of an *App* allele (11.1 weeks for *Npc1*^−/−^*/App*^*+/+*^, 10.4 weeks for *Npc1*^−/−^*/App*^+/−^, and 9.4 weeks for *Npc1*^−/−^*/App*^−/−^. *Npc1*^*+/+*^*/App*^*+/+*^).

## Conclusions

In summary, our work revealed that loss of APP function in the NPC brain exacerbates the pathogenic neuroinflammation that occurs prior to symptomatic onset, exerting a direct impact on the four major inflammatory pathways previously identified in this mouse model of NPC, namely activation of microglia, anti-viral response, activation of T-lymphocytes, and chemotaxis of T-lymphocytes. These findings shed new light on the function of APP as a cytoprotective modulator in the CNS, offering potential much-needed evidence-based therapies against NPC.

## Supplementary information

**Additional file 1: Figure S1.** Volcano plot representation of *Npc1*^*-/-*^ mouse cerebellar transcriptome. Vertical lines represent fold-change cutoff at -1.5 and 1.5, respectively (log2 scale). Horizontal line indicates *p*-value cutoff at *p* < 0.05 (-log scale). Red = differentially expressed transcripts. Black = non-significant transcripts.

**Additional file 2: Figure S2.** Volcano plot representations of *Npc1*^*+/+*^*/App*^*-/-*^ mouse cerebellar transcriptome. Vertical lines represent fold-change cutoff at -1.5 and 1.5, respectively (log2 scale). Horizontal line indicates *p*-value cutoff at *p* < 0.05 (-log scale). Red = differentially expressed transcripts. Black = non-significant transcripts.

**Additional file 3: Figure S3.** Volcano plot representations of *Npc1*^*-/-*^*/App*^*-/-*^ mouse cerebellar transcriptome. Vertical lines represent fold-change cutoff at -1.5 and 1.5, respectively (log2 scale). Horizontal line indicates *p*-value cutoff at *p* < 0.05 (-log scale). Red = differentially expressed transcripts. Black = non-significant transcripts.

**Additional file 4: Figure S4.** Loss of APP function results in the exacerbation of DEGs functionally related to the activation of microglia in *Npc1*^*-/-*^*/App*^*-/-*^ mouse cerebella. All differentially expressed genes (DEGs) are localized to their sub-cellular location. All plotted DEGs meet the significance cutoff of fold-change (absolute FC > 1.5) and *p*-value (*p* < 0.05). *Duplicate identifiers used for the same gene. A detailed key for IPA molecular shape, color, and interaction is provided in Fig. 2.

**Additional file 5: Figure S5.** Loss of APP function results in the exacerbation of DEGs functionally related to antiviral response in *Npc1*^*-/-*^*/App*^*-/-*^ mouse cerebella. All differentially expressed genes (DEGs) are localized to their sub-cellular location. All plotted DEGs meet the significance cutoff of fold-change (absolute FC > 1.5) and *p*-value (*p* < 0.05). *Duplicate identifiers used for the same gene. A detailed key for IPA molecular shape, color, and interaction is provided in Fig. 2.

**Additional file 6: Figure S6.** Loss of APP function results in the activation of the antimicrobial response pathway in *Npc1*^*-/-*^*/App*^*-/-*^ mouse cerebella. In *Npc1*^*-/-*^*/App*^*-/-*^ mouse cerebella, 83 genes related to antimicrobial response were differentially expressed when compared with wild-type littermates (*Npc1*^*+/+*^*/App*^*+/+*^). IPA *Upstream Analysis* further identified that 62 of these genes are IFN-γ-responsive and 44 are identified to be IFN-α-responsive. All differentially expressed genes (DEGs) are localized to their sub-cellular location. All plotted DEGs meet the significance cutoff of fold-change (absolute FC > 1.5) and *p*-value (*p* < 0.05). *Duplicate identifiers used for the same gene. A detailed key for IPA molecular shape, color, and interaction is provided in Fig. 2.

**Additional file 7: Figure S7.** Loss of APP function results in the exacerbation of DEGs functionally related to the activation of T-lymphocytes in *Npc1*^*-/-*^*/App*^*-/-*^ mouse cerebella. All differentially expressed genes (DEGs) are localized to their sub-cellular location. All plotted DEGs meet the significance cutoff of fold-change (absolute FC > 1.5) and *p*-value (*p* < 0.05). *Duplicate identifiers used for the same gene. A detailed key for IPA molecular shape, color, and interaction is provided in Fig. 2.

**Additional file 8: Figure S8.** Activation of T-lymphocyte co-stimulatory receptor CD28 in *Npc1*^*-/-*^*/App*^*-/-*^ mouse cerebella. All differentially expressed genes (DEGs) are localized to their sub-cellular location. All plotted DEGs meet the significance cutoff of fold-change (absolute FC > 1.5) and *p*-value (*p* < 0.05). *Duplicate identifiers used for the same gene. A detailed key for IPA molecular shape, color, and interaction is provided in Fig. 4.

**Additional file 9: Figure S9.** Loss of APP function results in the exacerbation of DEGs functionally related to the chemotaxis of T-lymphocytes in *Npc1*^*-/-*^*/App*^*-/-*^ mouse cerebella. All differentially expressed genes (DEGs) are localized to their sub-cellular location. All plotted DEGs meet the significance cutoff of fold-change (absolute FC > 1.5) and *p*-value (*p* < 0.05). *Duplicate identifiers used for same gene. A detailed IPA key for molecular shape, color and interaction is provided in Fig. 2.

**Additional file 10: Figure S10.** Loss of APP function results in the exacerbation of DEGs functionally related to the activation of antigen presenting cells in *Npc1*^*-/-*^*/App*^*-/-*^ mouse cerebella. All differentially expressed genes (DEGs) are localized to their sub-cellular location. All plotted DEGs meet the significance cutoff of fold-change (absolute FC > 1.5) and *p*-value (*p* < 0.05). *Duplicate identifiers used for the same gene. A detailed key for IPA molecular shape, color, and interaction is provided in Fig. 2.

**Additional file 11: Figure S11.** The activation of dendritic cells is implicated in the *Npc1*^*-/-*^*/App*^*-/-*^ mouse cerebella as a result of APP loss of function. All differentially expressed genes (DEGs) are localized to their sub-cellular location. All plotted DEGs meet the significance cutoff of fold-change (absolute FC > 1.5) and *p*-value (*p* < 0.05). *Duplicate identifiers used for the same gene. A detailed key for IPA molecular shape, color, and interaction is provided in Fig. 4.

**Additional file 12:** **Figure S12.** Activation of APC-associated co-stimulatory molecules is implicated in *Npc1*^*-/-*^*/App*^*-/-*^ mouse cerebella. In *Npc1*^*-/-*^*/App*^*-/-*^ mouse cerebella, 32 genes related to CD40, 12 genes related to ICAM1, and 6 genes related to CD86 were differentially expressed when compared with wildtype littermates (*Npc1*^*+/+*^*/App*^*+/+*^). All differentially expressed genes (DEGs) are localized to their sub-cellular location. All plotted DEGs meet the significance cutoff of fold-change (absolute FC > 1.5) and *p*-value (*p* < 0.05). *Duplicate identifiers used for the same gene. A detailed key for IPA molecular shape, color, and interaction is provided in Fig. 4.

**Additional file 13: Figure S13.** Pleotropic and variable cytokine/chemokine expressions in the terminal stage cerebella of *Npc1*^*-/-*^*/App*^*+/+*^, *Npc1*^*-/-*^*/App*^*+/-*^, and *Npc1*^*-/-*^*/App*^*-/-*^ compared with *Npc1*^*+/+*^*/App*^*+/+*^ and *Npc1*^*+/+*^*/App*^*-/-*^. Values are means ± SEM. *p < 0.05, **p < 0.01. * = compared with *Npc1*^*+/+*^*/App*^*+/+*^; ^ = compared with *Npc1*^*+/+*^*/App*^*-/-*^; # = compared with *Npc1*^*-/-*^*/App*^*+/+*^.

**Additional file 14: Figure S14.** Infiltration of CD3+ T cells in cerebellum. Immunohistochemical staining reveals the absence of CD3+ cells in the cerebellum of mice of wildtype, *Npc1*^*-/-*^, *App*^*-/-*^ and *App*^*-/-*^*/Npc1*^*-/-*^ mice at 3 weeks of age. (A-C) *Npc1*^*+/+*^*/App*^*+/+*^ mice. (D-F) *Npc1*^*-/-*^*/App*^*+/+*^ mice. (G-I) *Npc1*^*+/+*^*/App*^*-/-*^ mice. (J-L) *App*^*-/-*^*/Npc1*^*-/-*^ mice. g: granular layer of the cerebellum; m: molecular layer of the cerebellum. White arrows show areas of stained patterns artifactual in nature, as they appear in all genotypes and all ages tested.

**Additional file 15: Table S1.** Number of cerebellar samples for multiplex cytokine/chemokine analysis.

## Abbreviations

aFC: Absolute fold-change
AICD: Amyloid precursor protein intracellular domain
APP: Amyloid precursor protein
Aβ: Beta-amyloid peptide
CCL11: Eotaxin
CNS: Central nervous system
DEGs: Differentially expressed genes
FDR: False discovery rate
G-CSF: Granulocyte colony-stimulating factor
GM-CSF: Granulocyte-macrophage colony-stimulating factor
GSEA: Gene set enrichment analysis
IFN-α: Interferon-alpha
IFN-γ: Interferon-gamma
IL-10: Interleukin-10
IL-12/p40: Interleukin-12
IL-12/p70: Interleukin-12
IL-13: Interleukin-13
IL-15: Interleukin-15
IL-17: Interleukin-17
IL-1α: Interleukin-1α
IL-1β: Interleukin-1β
IL-2: Interleukin-2
IL-3: Interleukin-3
IL-4: Interleukin-4
IL-5: Interleukin-5
IL-6: Interleukin-6
IL-7: Interleukin-7
IL-9: Interleukin-9
IP-10/CXCL10: Interferon-gamma-induced protein 10
IPA: Ingenuity Pathway Analysis
KC/CXCL1: Keratinocyte chemoattractant
LIF: Leukemia inhibitory factor
LIX/CXCL5: Lipopolysaccharide-inducible CXC chemokine
MCP-1/CCL2: Monocyte chemoattractant protein-1
M-CSF: Macrophage colony-stimulating factor
MIG/CXCL9: Monokine induced by gamma interferon
MIP-1α/CCL3: Macrophage inflammatory protein-1α
MIP-1β/CCL4: Macrophage inflammatory protein-1β
MIP-2/CXCL2: Macrophage inflammatory protein-2
MSigDB: Molecular signature database
NES: Normalized enrichment score
NPC: Niemann-Pick disease type C
RANTES/CCL5: Regulated on activation normal T cell expressed and secreted
TNF-α: Tumor necrosis factor alpha
TRs: transcript reads
VEGF: Vascular endothelial growth factor
