# Supplementary figures and images for: Loss of amyloid precursor protein exacerbates early inflammation in Niemann-Pick disease type C
Source: J Neuroinflammation. 2019 Dec 17;16:269. doi: 10.1186/s12974-019-1663-5 (PMC6918596; doi:10.1186/s12974-019-1663-5)

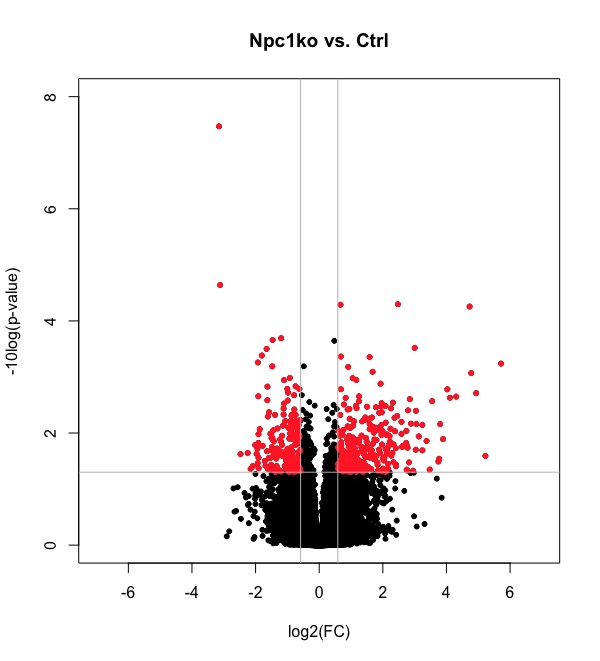

Supplement: Supplementary file 1 — Additional file 1: Figure S1. Volcano plot representation of Npc1-/- mouse cerebellar transcriptome. Vertical lines represent fold-change cutoff at -1.5 and 1.5, respectively (log2 scale). Horizontal line indicates p-value cutoff at p < 0.05 (-log scale). Red = differentially expressed transcripts. Black = non-significant transcripts. [file 12974_2019_1663_MOESM1_ESM.jpg]

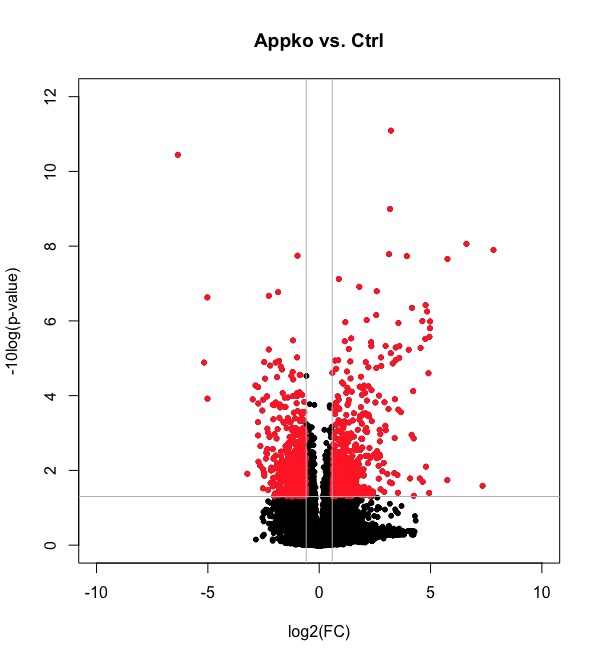

Supplement: Supplementary file 2 — Additional file 2: Figure S2. Volcano plot representations of Npc1+/+/App-/- mouse cerebellar transcriptome. Vertical lines represent fold-change cutoff at -1.5 and 1.5, respectively (log2 scale). Horizontal line indicates p-value cutoff at p < 0.05 (-log scale). Red = differentially expressed transcripts. Black = non-significant transcripts. [file 12974_2019_1663_MOESM2_ESM.jpg]

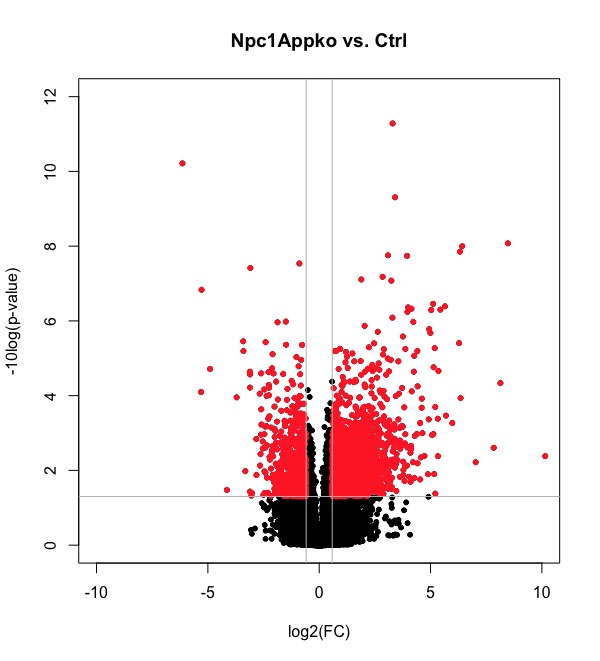

Supplement: Supplementary file 3 — Additional file 3: Figure S3. Volcano plot representations of Npc1-/-/App-/- mouse cerebellar transcriptome. Vertical lines represent fold-change cutoff at -1.5 and 1.5, respectively (log2 scale). Horizontal line indicates p-value cutoff at p < 0.05 (-log scale). Red = differentially expressed transcripts. Black = non-significant transcripts. [file 12974_2019_1663_MOESM3_ESM.jpg]

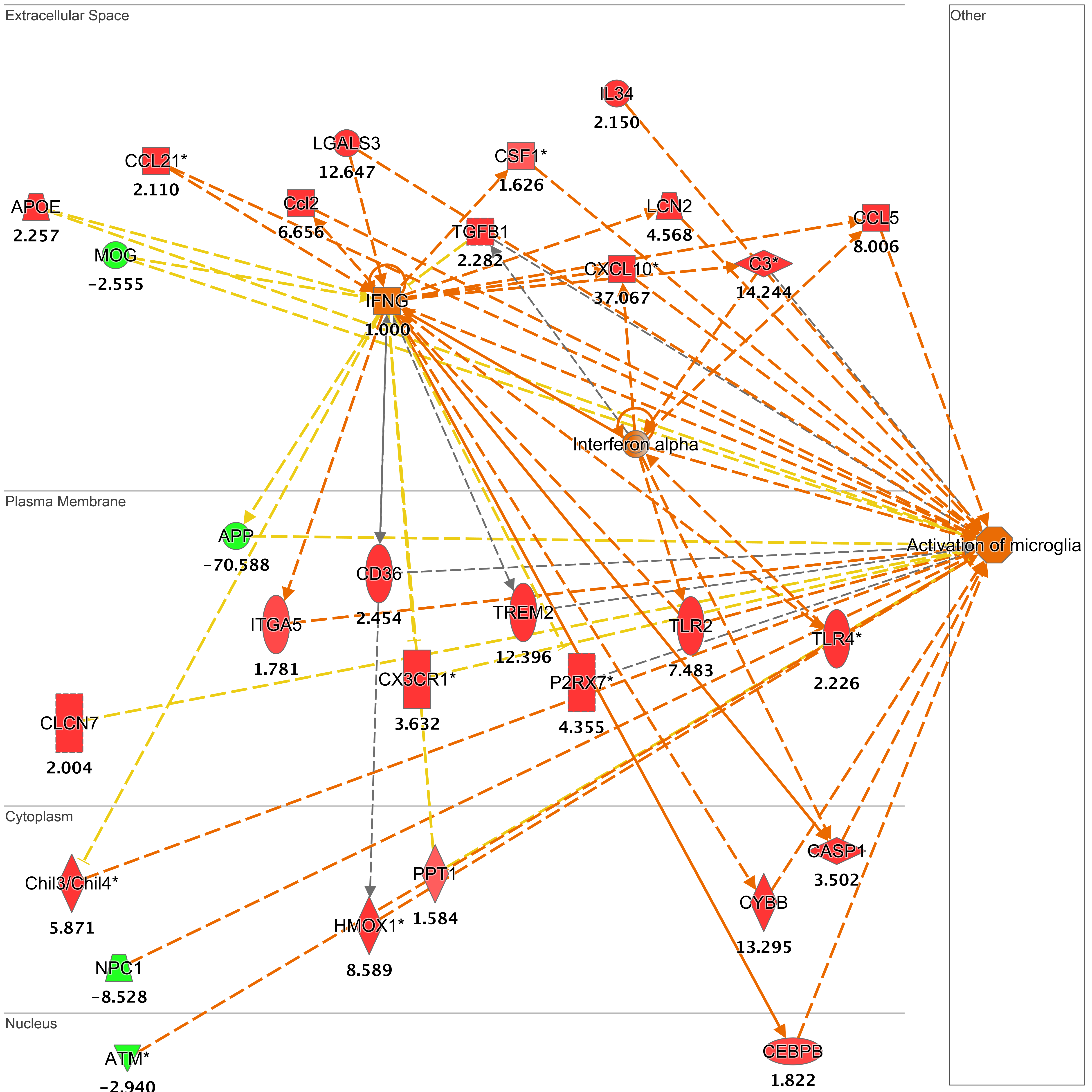

Supplement: Supplementary file 4 — Additional file 4: Figure S4. Loss of APP function results in the exacerbation of DEGs functionally related to the activation of microglia in Npc1-/-/App-/- mouse cerebella. All differentially expressed genes (DEGs) are localized to their sub-cellular location. All plotted DEGs meet the significance cutoff of fold-change (absolute FC > 1.5) and p-value (p < 0.05). *Duplicate identifiers used for the same gene. A detailed key for IPA molecular shape, color, and interaction is provided in Fig. 2. [file 12974_2019_1663_MOESM4_ESM.tif]

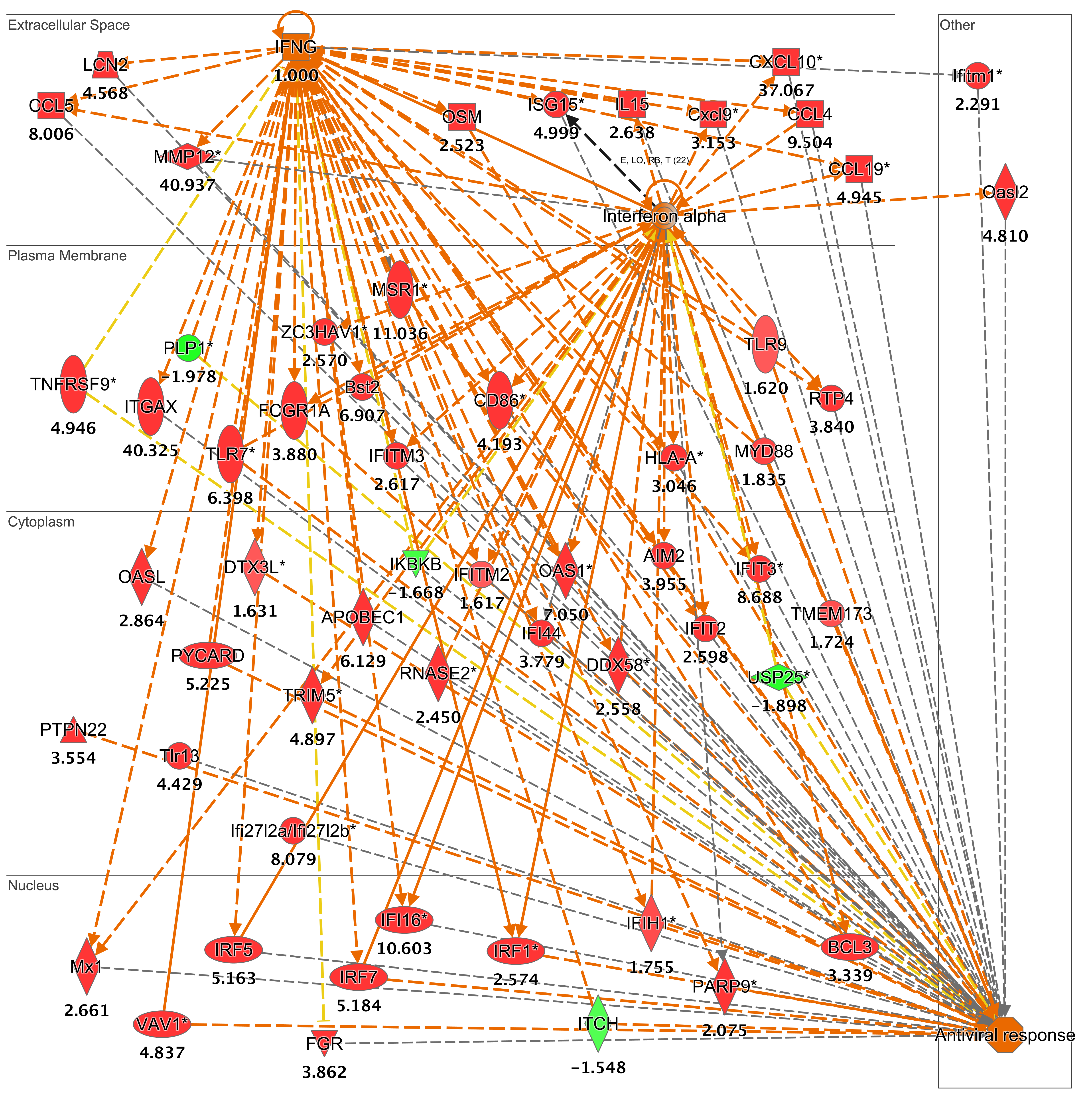

Supplement: Supplementary file 5 — Additional file 5: Figure S5. Loss of APP function results in the exacerbation of DEGs functionally related to antiviral response in Npc1-/-/App-/- mouse cerebella. All differentially expressed genes (DEGs) are localized to their sub-cellular location. All plotted DEGs meet the significance cutoff of fold-change (absolute FC > 1.5) and p-value (p < 0.05). *Duplicate identifiers used for the same gene. A detailed key for IPA molecular shape, color, and interaction is provided in Fig. 2. [file 12974_2019_1663_MOESM5_ESM.tif]

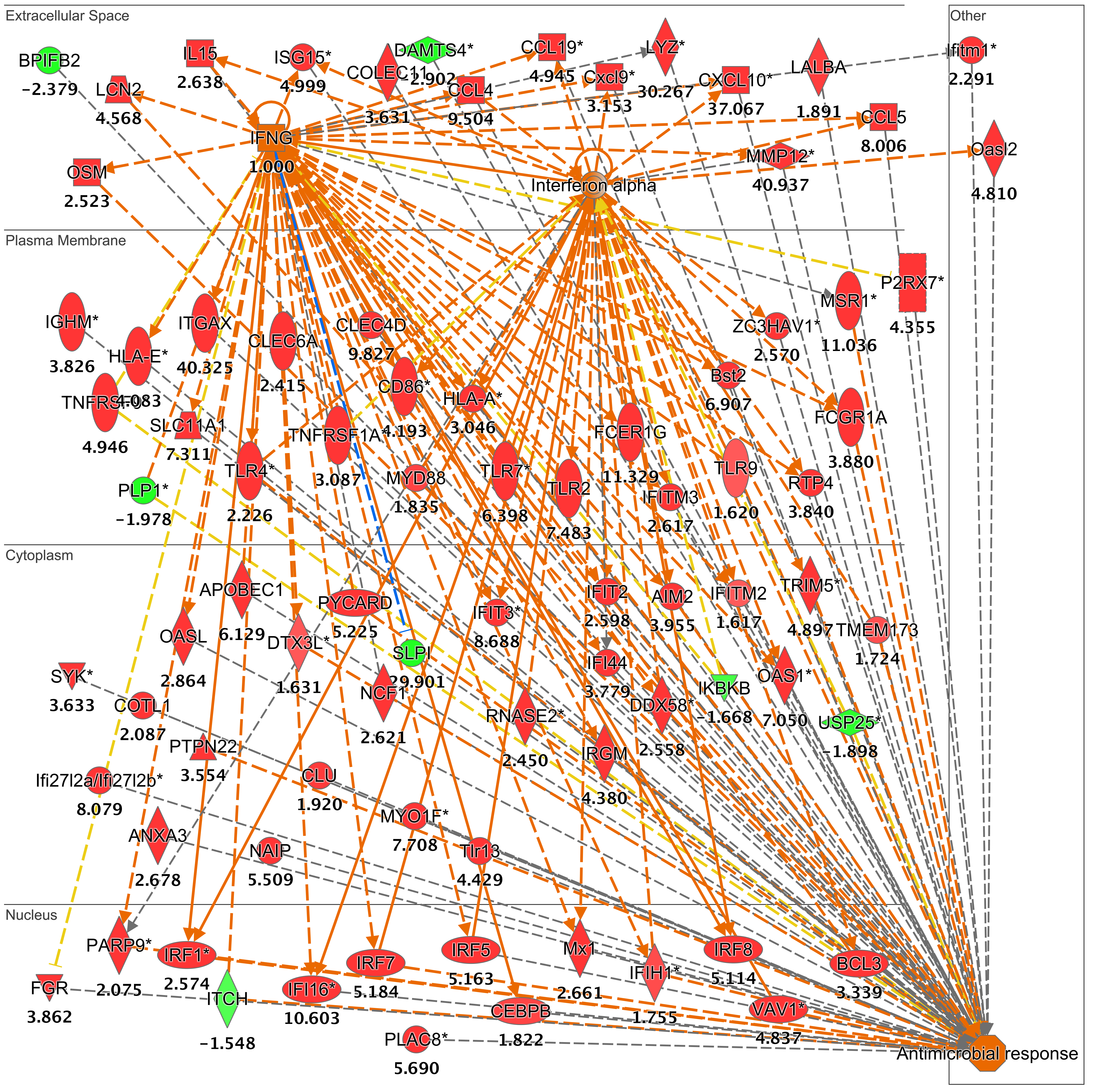

Supplement: Supplementary file 6 — Additional file 6: Figure S6. Loss of APP function results in the activation of the antimicrobial response pathway in Npc1-/-/App-/- mouse cerebella. In Npc1-/-/App-/- mouse cerebella, 83 genes related to antimicrobial response were differentially expressed when compared with wild-type littermates (Npc1+/+/App+/+). IPA Upstream Analysis further identified that 62 of these genes are IFN-γ-responsive and 44 are identified to be IFN-α-responsive. All differentially expressed genes (DEGs) are localized to their sub-cellular location. All plotted DEGs meet the significance cutoff of fold-change (absolute FC > 1.5) and p-value (p < 0.05). *Duplicate identifiers used for the same gene. A detailed key for IPA molecular shape, color, and interaction is provided in Fig. 2. [file 12974_2019_1663_MOESM6_ESM.tif]

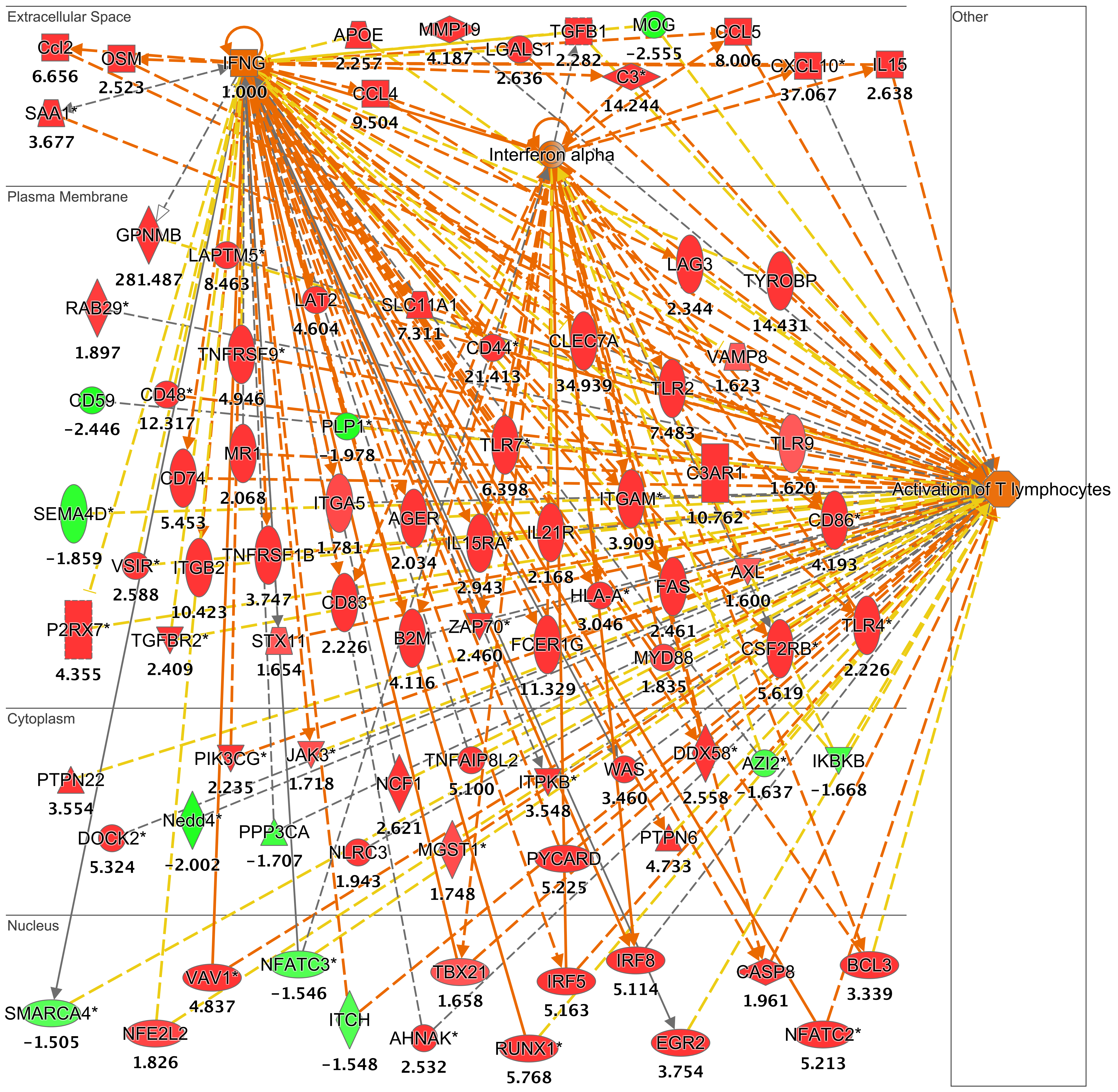

Supplement: Supplementary file 7 — Additional file 7: Figure S7. Loss of APP function results in the exacerbation of DEGs functionally related to the activation of T-lymphocytes in Npc1-/-/App-/- mouse cerebella. All differentially expressed genes (DEGs) are localized to their sub-cellular location. All plotted DEGs meet the significance cutoff of fold-change (absolute FC > 1.5) and p-value (p < 0.05). *Duplicate identifiers used for the same gene. A detailed key for IPA molecular shape, color, and interaction is provided in Fig. 2. [file 12974_2019_1663_MOESM7_ESM.tif]

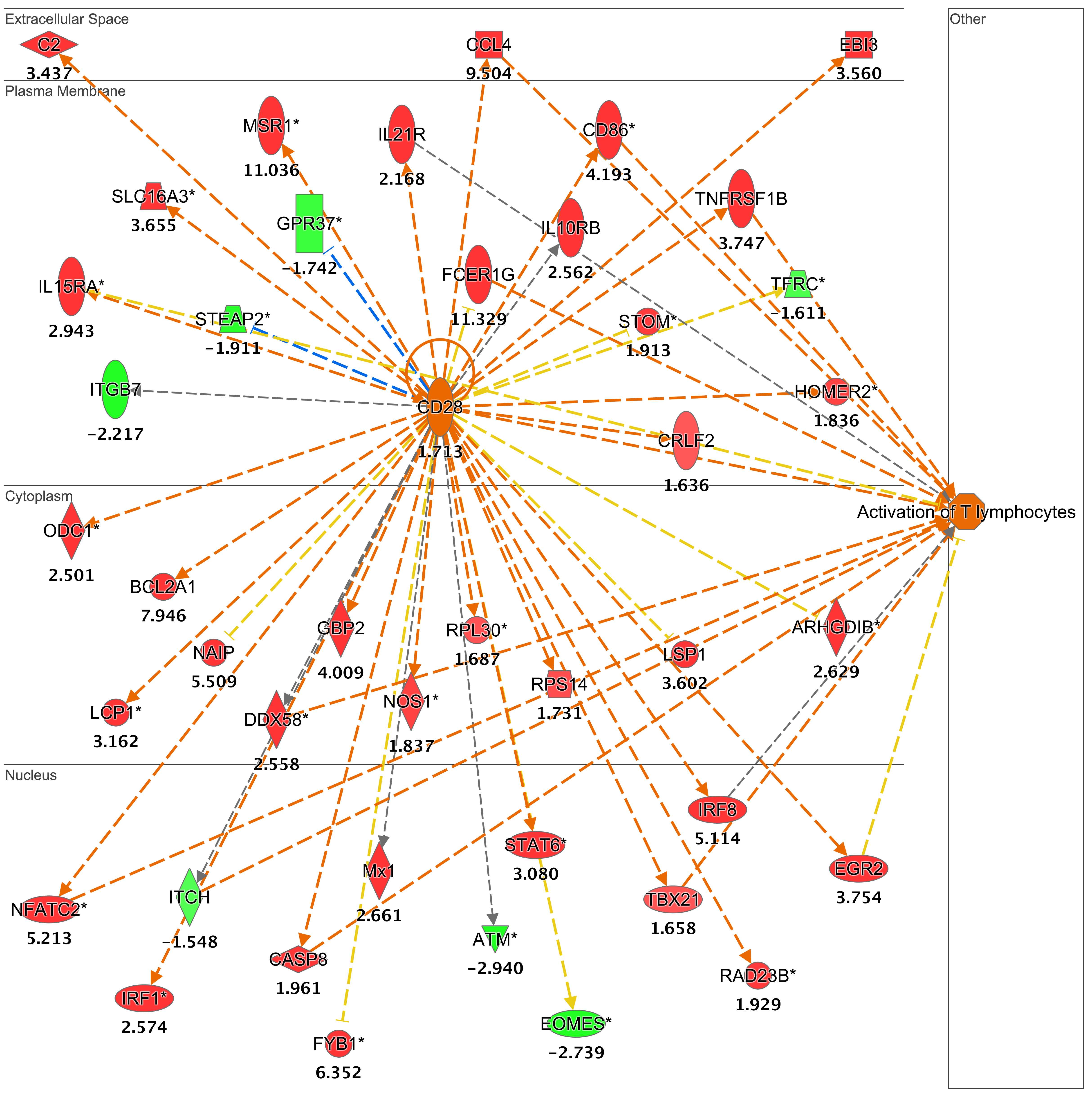

Supplement: Supplementary file 8 — Additional file 8: Figure S8. Activation of T-lymphocyte co-stimulatory receptor CD28 in Npc1-/-/App-/- mouse cerebella. All differentially expressed genes (DEGs) are localized to their sub-cellular location. All plotted DEGs meet the significance cutoff of fold-change (absolute FC > 1.5) and p-value (p < 0.05). *Duplicate identifiers used for the same gene. A detailed key for IPA molecular shape, color, and interaction is provided in Fig. 4. [file 12974_2019_1663_MOESM8_ESM.jpg]

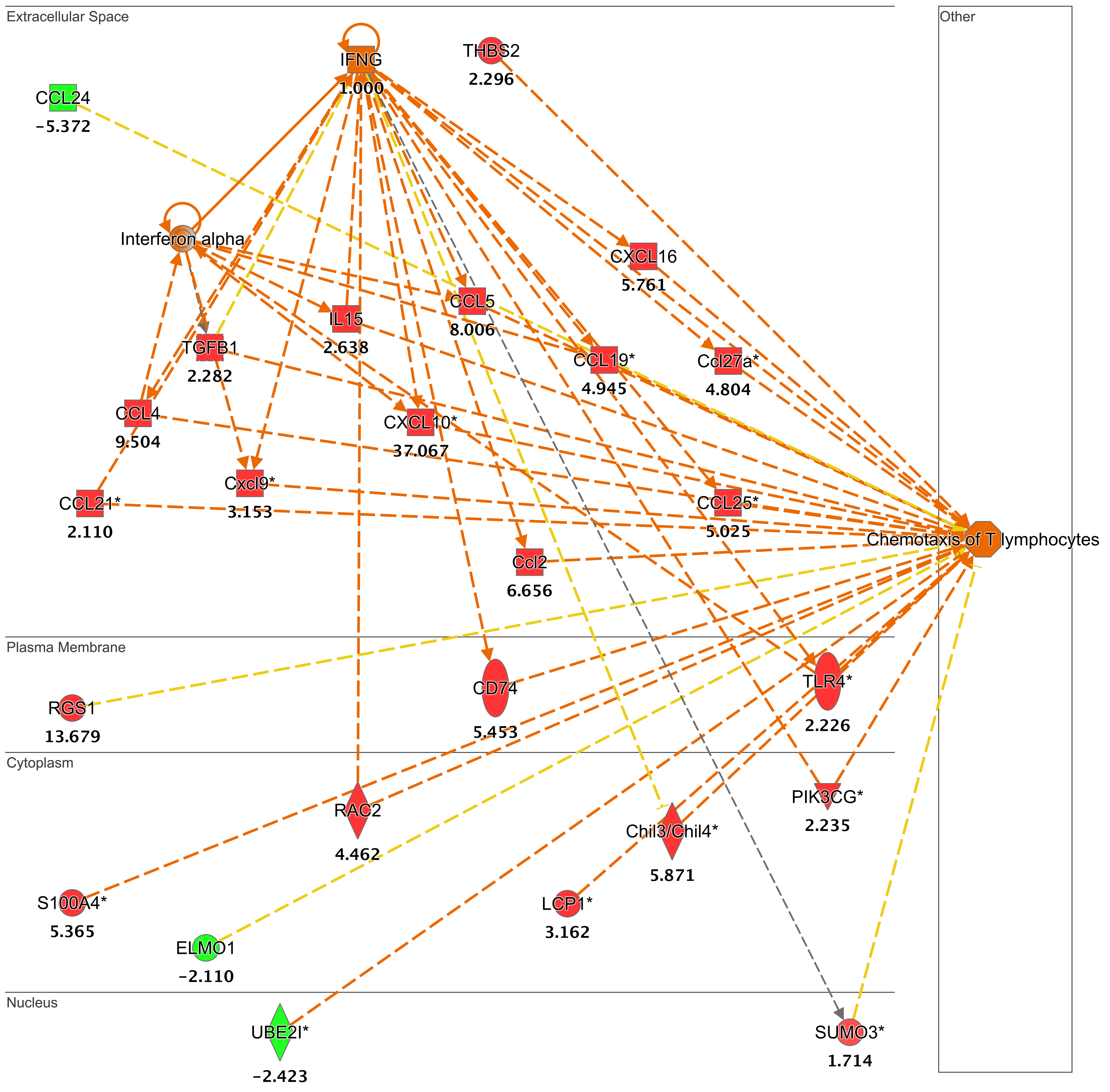

Supplement: Supplementary file 9 — Additional file 9: Figure S9. Loss of APP function results in the exacerbation of DEGs functionally related to the chemotaxis of T-lymphocytes in Npc1-/-/App-/- mouse cerebella. All differentially expressed genes (DEGs) are localized to their sub-cellular location. All plotted DEGs meet the significance cutoff of fold-change (absolute FC > 1.5) and p-value (p < 0.05). *Duplicate identifiers used for same gene. A detailed IPA key for molecular shape, color and interaction is provided in Fig. 2. [file 12974_2019_1663_MOESM9_ESM.tiff]

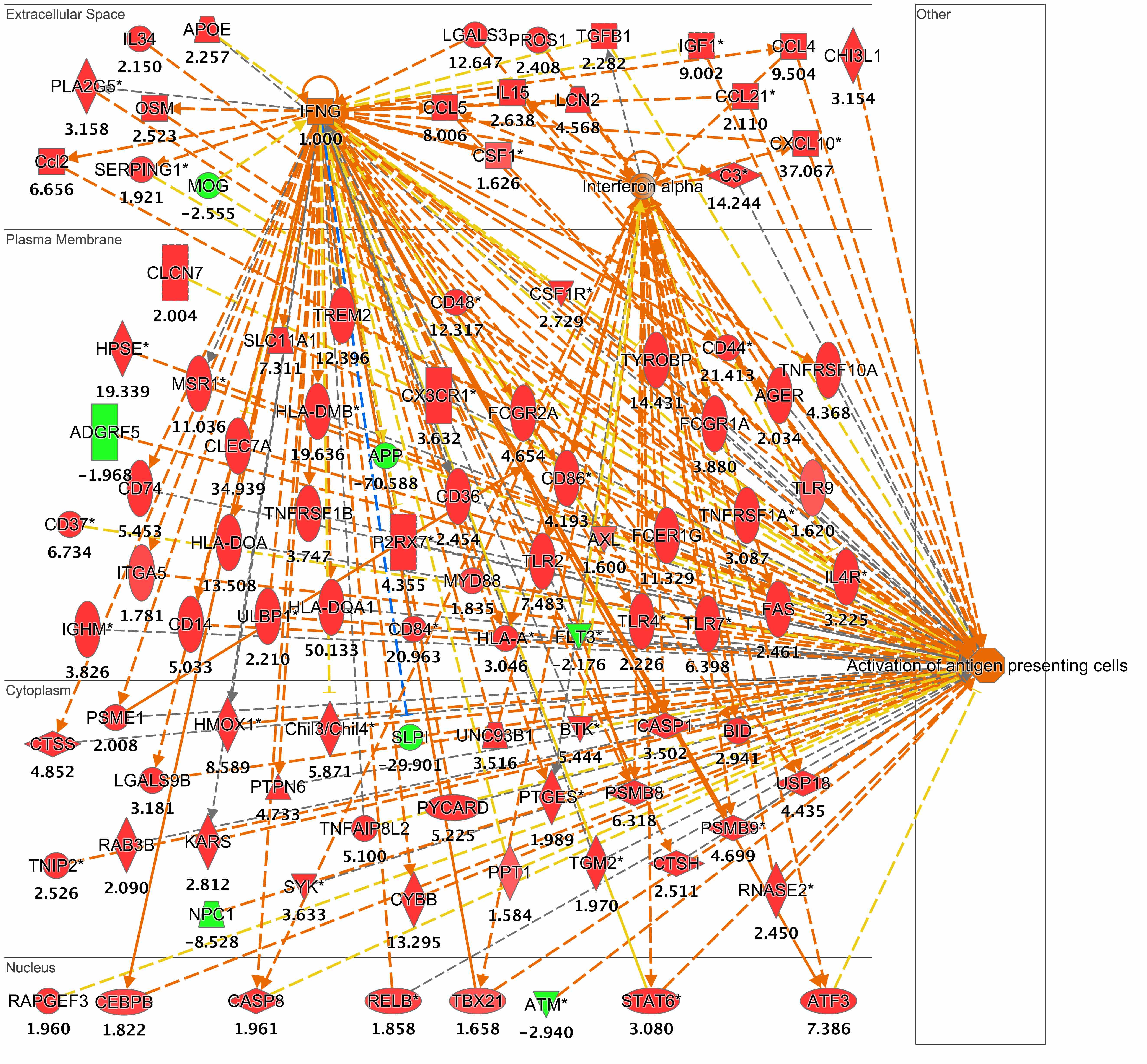

Supplement: Supplementary file 10 — Additional file 10: Figure S10. Loss of APP function results in the exacerbation of DEGs functionally related to the activation of antigen presenting cells in Npc1-/-/App-/- mouse cerebella. All differentially expressed genes (DEGs) are localized to their sub-cellular location. All plotted DEGs meet the significance cutoff of fold-change (absolute FC > 1.5) and p-value (p < 0.05). *Duplicate identifiers used for the same gene. A detailed key for IPA molecular shape, color, and interaction is provided in Fig. 2. [file 12974_2019_1663_MOESM10_ESM.jpg]

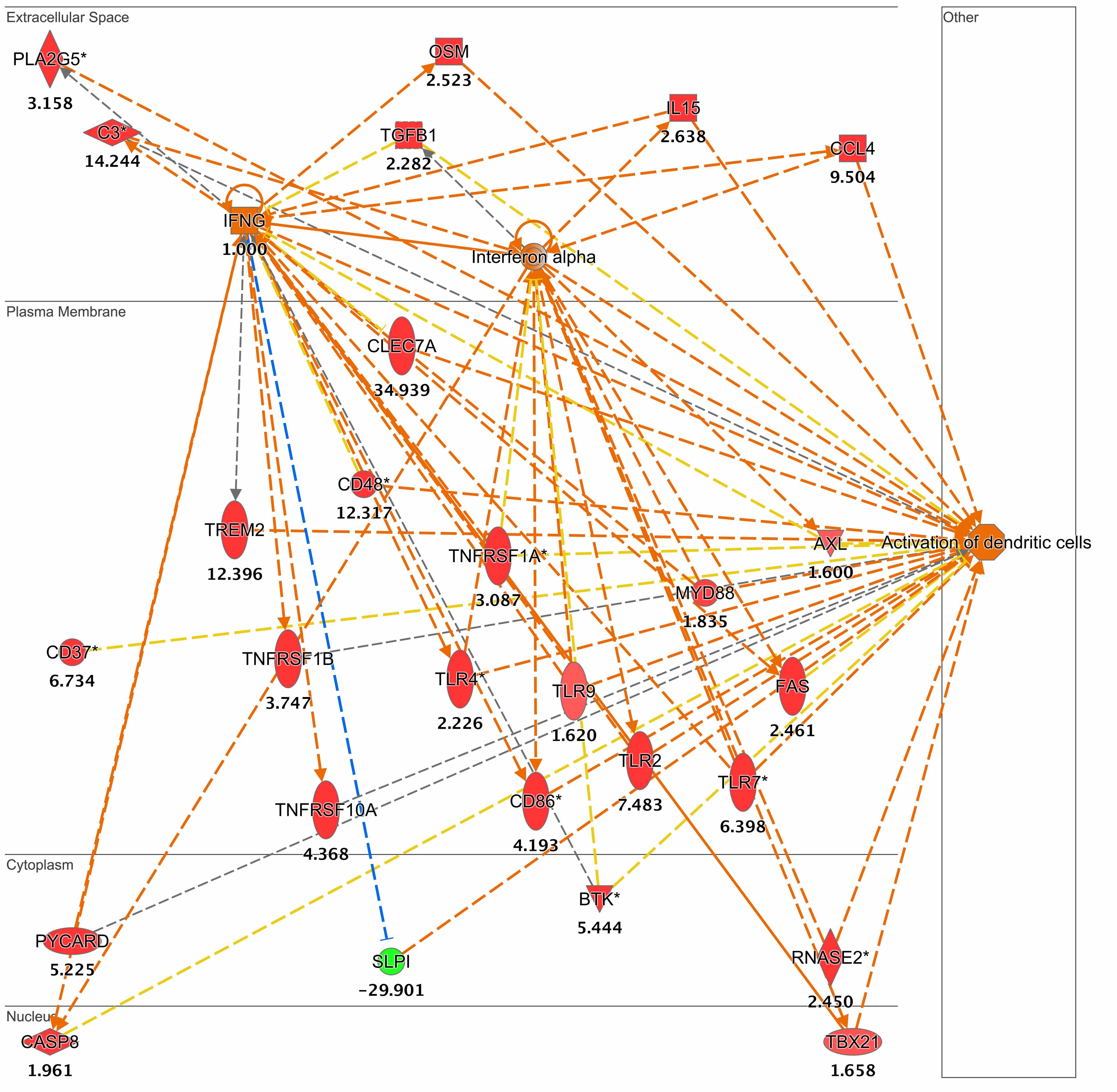

Supplement: Supplementary file 11 — Additional file 11: Figure S11. The activation of dendritic cells is implicated in the Npc1-/-/App-/- mouse cerebella as a result of APP loss of function. All differentially expressed genes (DEGs) are localized to their sub-cellular location. All plotted DEGs meet the significance cutoff of fold-change (absolute FC > 1.5) and p-value (p < 0.05). *Duplicate identifiers used for the same gene. A detailed key for IPA molecular shape, color, and interaction is provided in Fig. 4. [file 12974_2019_1663_MOESM11_ESM.jpg]

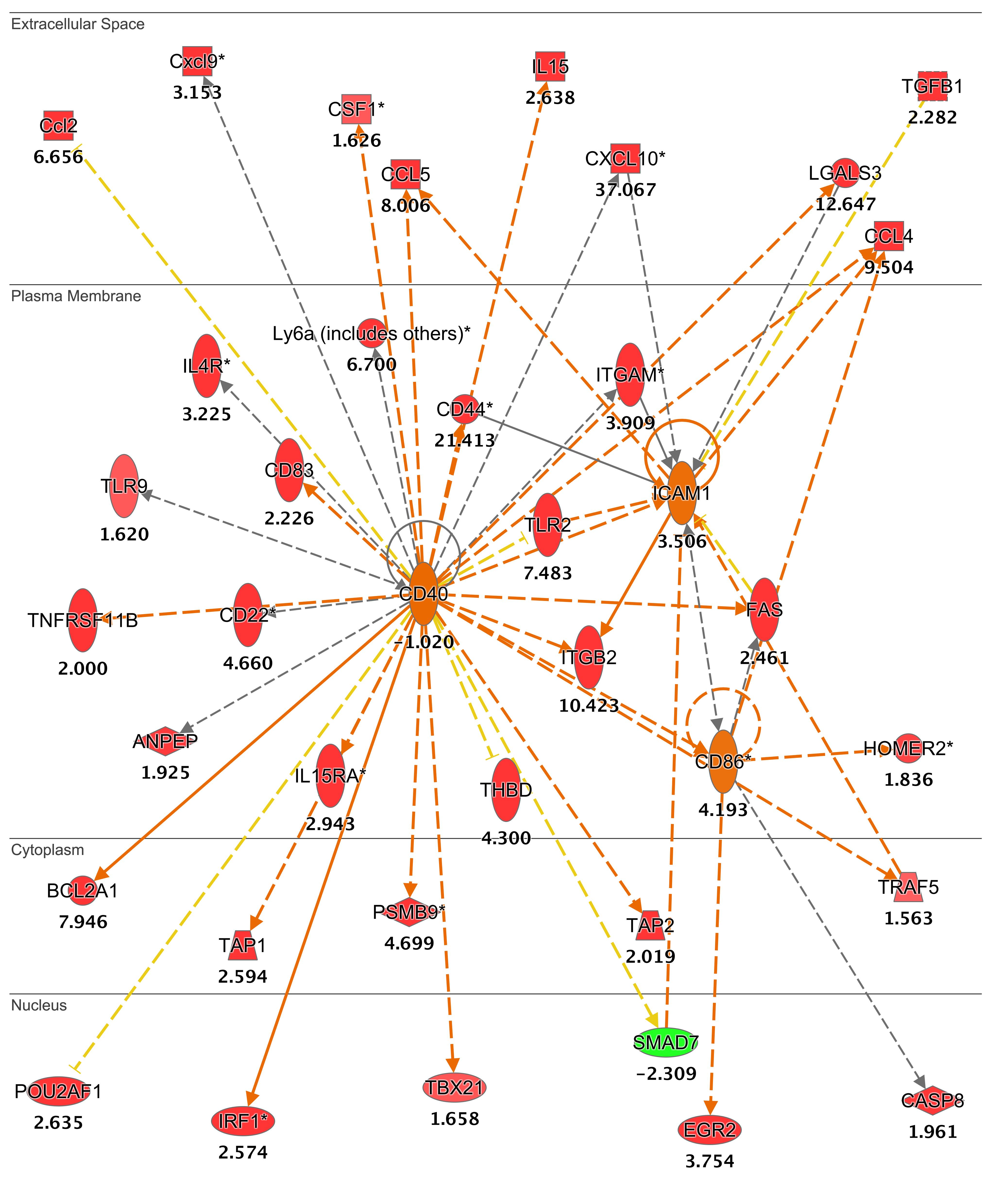

Supplement: Supplementary file 12 — Additional file 12: Figure S12. Activation of APC-associated co-stimulatory molecules is implicated in Npc1-/-/App-/- mouse cerebella. In Npc1-/-/App-/- mouse cerebella, 32 genes related to CD40, 12 genes related to ICAM1, and 6 genes related to CD86 were differentially expressed when compared with wildtype littermates (Npc1+/+/App+/+). All differentially expressed genes (DEGs) are localized to their sub-cellular location. All plotted DEGs meet the significance cutoff of fold-change (absolute FC > 1.5) and p-value (p < 0.05). *Duplicate identifiers used for the same gene. A detailed key for IPA molecular shape, color, and interaction is provided in Fig. 4. [file 12974_2019_1663_MOESM12_ESM.jpg]

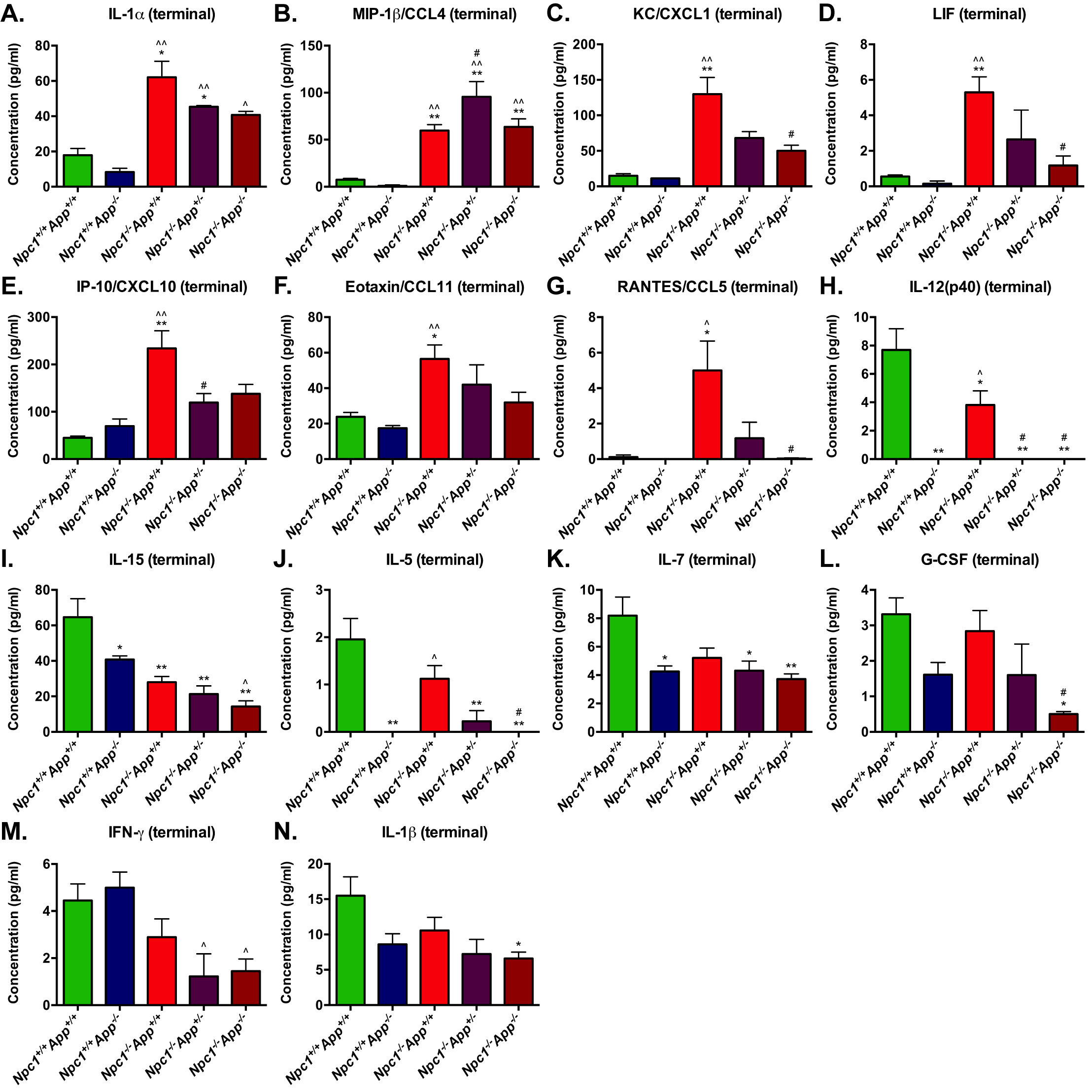

Supplement: Supplementary file 13 — Additional file 13: Figure S13. Pleotropic and variable cytokine/chemokine expressions in the terminal stage cerebella of Npc1-/-/App+/+, Npc1-/-/App+/-, and Npc1-/-/App-/- compared with Npc1+/+/App+/+ and Npc1+/+/App-/-. Values are means ± SEM. *p < 0.05, **p < 0.01. * = compared with Npc1+/+/App+/+; ^ = compared with Npc1+/+/App-/-; # = compared with Npc1-/-/App+/+. [file 12974_2019_1663_MOESM13_ESM.tiff]

DAPI

CD3

DAPI + CD3

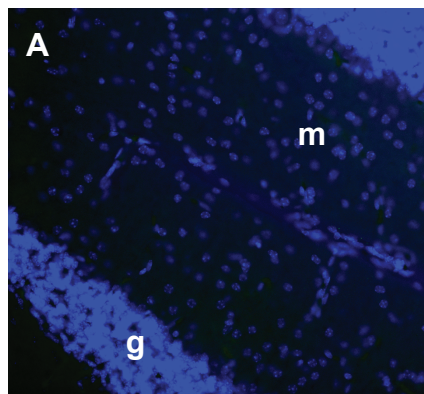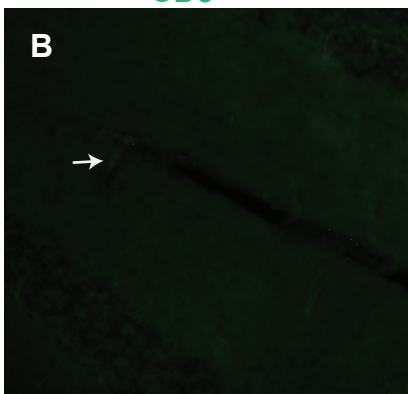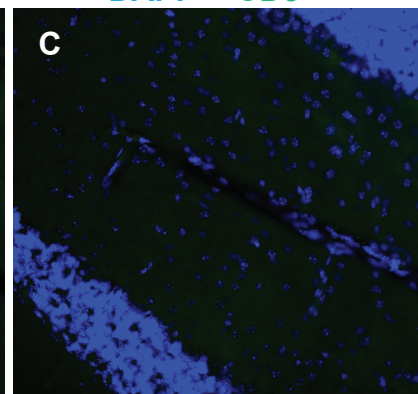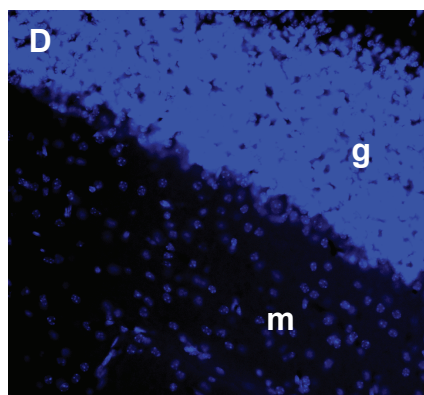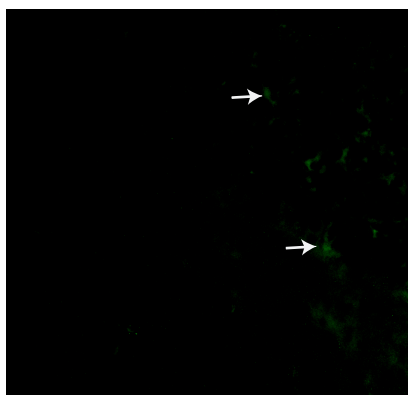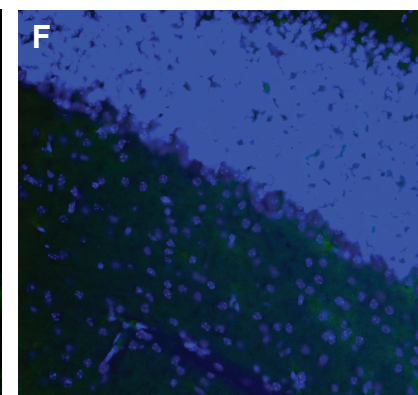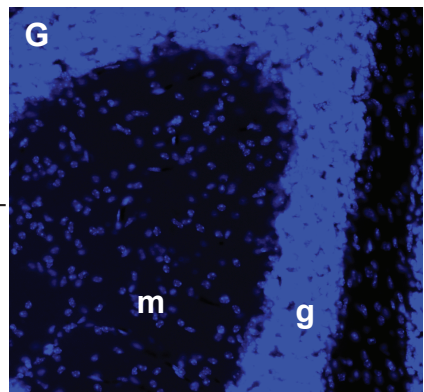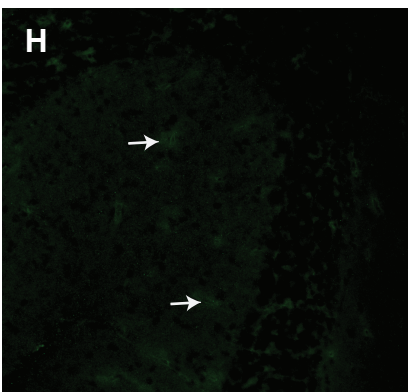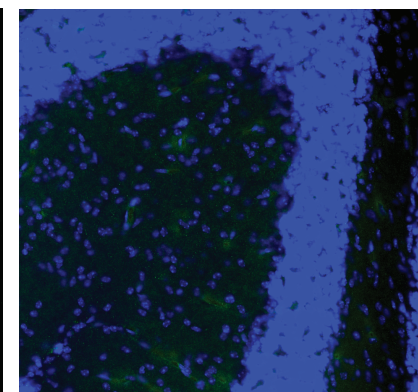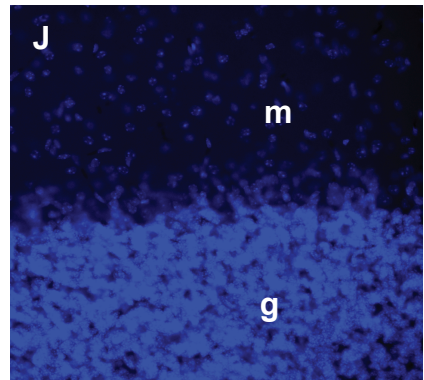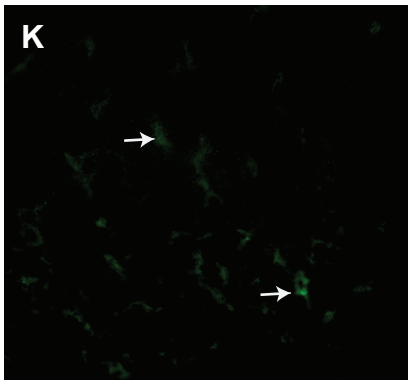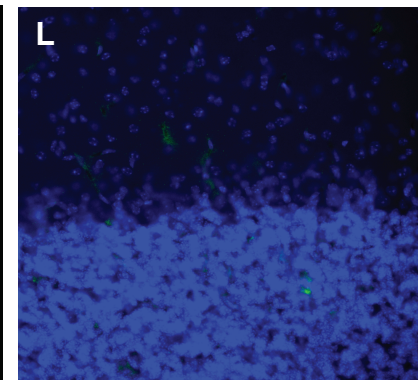

Npc+/+/App+/+  
3 weeks

Npc1-/-/App+/+  
3 weeks

Npc1+/+/App1-/-  
3 weeks

Npc1-/-/App-/-  
3 weeks

Supplement: Supplementary file 14 — Additional file 14: Figure S14. Infiltration of CD3+ T cells in cerebellum. Immunohistochemical staining reveals the absence of CD3+ cells in the cerebellum of mice of wildtype, Npc1-/-, App-/- and App-/-/Npc1-/- mice at 3 weeks of age. (A-C) Npc1+/+/App+/+ mice. (D-F) Npc1-/-/App+/+ mice. (G-I) Npc1+/+/App-/- mice. (J-L) App-/-/Npc1-/- mice. g: granular layer of the cerebellum; m: molecular layer of the cerebellum. White arrows show areas of stained patterns artifactual in nature, as they appear in all genotypes and all ages tested. [file 12974_2019_1663_MOESM14_ESM.pdf]
