# Supplementary material for: Loss of amyloid precursor protein exacerbates early inflammation in Niemann-Pick disease type C
Source: J Neuroinflammation. 2019 Dec 17;16:269. doi: 10.1186/s12974-019-1663-5 (PMC6918596; doi:10.1186/s12974-019-1663-5)
Supplement: Supplementary file 15 — Additional file 15: Table S1. Number of cerebellar samples for multiplex cytokine/chemokine analysis. [file 12974_2019_1663_MOESM15_ESM.docx]

| Genotype | # of 3-week samples | # of terminal stage samples |
| --- | --- | --- |
| *Npc1^+/+^/App^+/+^* | 4 | 4 |
| *Npc1^+/+^/App^-/-^* | 5 | 4 |
| *Npc1^-/-^/App^+/+^* | 5 | 6 |
| *Npc1^-/-^/App^+/-^* | 4 | 4 |
| *Npc1^-/-^/App^-/-^* | 5 | 4 |

**Additional file 15: Table S1.** Number of cerebellar samples for Multiplex Cytokine/Chemokine analysis
